# Meteorological factors associated with the timing and abundance of *Hymenoscyphus fraxineus* spore release

**DOI:** 10.1007/s00484-021-02211-z

**Published:** 2021-11-11

**Authors:** Paul Burns, Volkmar Timmermann, Jon M. Yearsley

**Affiliations:** 1 College of Engineering, Mathematics and Physical Sciences, Harrison Building, Streatham Campus, University of Exeter, North Park Rd, Exeter, EX4 4QF UK; 2 Division of Biotechnology and Plant Health, Norwegian Institute of Bioeconomy Research (NIBIO), P.O. Box. 115, 1431 Ås, Norway; 3 School of Biology & Environmental Science & UCD Earth Institute, University College Dublin, Belfield, Dublin 4, Ireland

**Keywords:** Fungal-pathogen, Spores, Meteorology, GAMs, Monte-Carlo

## Abstract

The ascomycete *Hymenoscyphus fraxineus* has spread across most of the host range of European ash with a high level of mortality, causing important economic, cultural and environmental effects. We present a novel method combining a Monte-Carlo approach with a generalised additive model that confirms the importance of meteorology to the magnitude and timing of *H. fraxineus* spore emissions. The variability in model selection and the relative degree to which our models are over- or under-fitting the data has been quantified. We find that both the daily magnitude and timing of spore emissions are affected by meteorology during and prior to the spore emission diurnal peak. We found the daily emission magnitude has the strongest associations to weekly average net radiation and leaf moisture before the emission, soil temperature during the day before emission and net radiation during the spore emission. The timing of the daily peak in spore emissions has the strongest associations to net radiation both during spore emission and in the day preceding the emission. The seasonal peak in spore emissions has a near-exponential increase/decrease, and the mean daily emission peak is approximately Gaussian.

**Supplementary Information:**

The online version contains supplementary material available at 10.1007/s00484-021-02211-z.

## Introduction

Fungal pathogens are a natural part of the biosphere and need to be properly understood and managed to ensure that agriculture and forestry are both efficient and sustainable (e.g. Dean et al. 2012). The present study focuses on the fungal pathogen *Hymenoscyphus fraxineus* (the causative agent of ash dieback, Baral et al. 2014; CABI 2019), which, like many other fungal pathogens, spreads to new host regions by dispersal of spores through the atmosphere. In particular, we focus on the diurnal timing and total quantity of *H. fraxineus* spore release.

Fruit bodies of *H. fraxineus* form during summer on leaf litter of overwintered ash petioles and rachises that have been infected in the previous year (Gross et al. 2014). The wind-dispersed ascospores are released from the fruit bodies and infect ash leaves during the vegetation period (Timmermann et al. 2011; Hietala et al. 2013; Chandelier et al. 2014), causing necroses and wilting of the leaves. Before leaf fall, the fungal mycelium grows through rachises and petioles into the shoots and branches and finally, during the dormancy period in winter, into the stem of the tree (Kirisits & Cech, 2009). Necroses in the tree tissue block water transport and lead to wilting and dieback of branches and may be fatal, often in combination with other factors such as root rot (e.g. *Armillaria*) or drought. Furthermore, the fungal spores can attack the root collar directly through lenticels in the stem (Nemesio-Gorriz et al. 2019), causing collar lesions and necroses in the stem, blocking water transport and thereby likely weakening the root system so that other, secondary pathogens, such as *Armillaria*, can infect the root system (Husson et al. 2012; Chandelier et al. 2016). A third infection pathway may be water-dispersed conidia (asexual spores) that enter the root system of ash trees through the soil (Fones et al. 2016).

The ascomycete *H. fraxineus* has now spread across most of the host range of European ash (*Fraxinus excelsior*) with a high level of mortality, causing important economic, cultural and environmental effects (European Commission 2013). In Norway, the annual mean distance of spread of the pathogen along the Norwegian west coast has been shown to be 51 km (Solheim and Hietala 2017). By 2018, *H. fraxineus* had spread through the entire natural distribution range of European ash in Norway (Timmermann et al. 2019; A. Hietala and H. Solheim, pers. comm.), only 12 years after its estimated introduction into the country.

The spores of *H. fraxineus* are similar to those of many other fungal pathogens such as wheat rust (*Puccinia graminis f.* sp. tritici). Race Ug99 of wheat rust threatens 90% of wheat production globally and so is recognised as a major threat to world food security (Dean et al. 2012). Consideration should be given to the possibility of pathogens changing (or jumping) hosts, increasing the threat posed by species such as *H. fraxineus*. This pathogen was likely introduced to Europe with ornamental trees from Asia, where it is regarded as a more or less harmless endophytic or only slightly parasitic fungus in leaves of its original host, Manchurian ash (*F. mandshurica*) (Cleary et al. 2016; Drenkhan et al. 2017). It first became a lethal pathogen when changing to a new host, European ash, a close relative of Manchurian ash but with a different distribution range. Increasing demand for supply and an increasingly variable climate demand an ever more efficient and resilient industry, which is reflected in the aims of the European Commission (European Commission 2013). In order to effectively manage fungal pathogens, it is important that we fully understand them and are able to predict their probable movements and impacts.

Like any airborne pathogen, the spread of *H. fraxineus* spores to new host areas can be divided into three steps: details of spore emission, the ability of spores to survive Earth’s atmosphere and on-host infection processes (e.g. Gross et al. 2014). Much is still unknown about the biology of *H. fraxineus* (e.g. Sansford 2013), which includes the pathogen’s ability to reproduce. The CABI datasheet provides a good overview of what’s known about the ecology/biology of the pathogen (CABI 2019). This work focuses on improving understanding of the emission of spores by *H. fraxineus*. Previous research provides important insights into *H. fraxineus* spore emission processes. Timmermann et al. (2011) noted that the observed early morning peak in spore emissions suggests that *H. fraxineus* actively emits (or ejects) its spores. This is in contrast to fungi with more passive spore emission, such as *Blumeria graminis* and *Puccinia striiformis*, that have a peak emission around mid-afternoon when wind currents and gusts are generally most intense (West et al. 2008). Most ascomycetes that actively emit their spores use a ‘water-cannon’ mechanism, which relies on the availability of moisture (Ingold, 1999). However, unknowns include the exact moisture requirement, the timescale required to accumulate enough water and the exact method of water absorption.

Spore maturation most likely occurs during the day preceding the morning peak emission period (Timmermann et al., 2011). If this is the case then environmental conditions (e.g. moisture and temperature) during the day prior to emission are likely to have some effect on the spore emission. Morning dew and moisture may protect the ascospores from desiccation during the subsequent infection process and may stimulate their germination (Timmermann et al. 2011). Sub-zero ground temperatures may turn dewfall into frost, which can be expected to hinder fungal spore emissions. In all cases, the details remain unclear, for example, what moisture and temperature levels are required? What types of moisture (ground, leaf, water vapour, rainfall) are most important? Is spore maturation (and fungal activity) affected by longer-term moisture and temperature trends?

The effects of meteorology on spore emission processes remain to be investigated (Timmermann et al. 2011). High winds and turbulence act to ventilate Earth’s atmospheric boundary layer (ABL), mixing aerosols (including fungal spores) to greater heights and so reducing near-ground aerosol concentrations (e.g. Dacre et al. 2007). On the other hand, winds may aid spore suspension, especially in sheltered forest locations. In still air spores will fall out of suspension at their ‘settling velocities’ (e.g. Di-Giovanni et al. 1995). Rainfall will ‘wash-out’ spores from the atmosphere (e.g. Aylor 2003) reducing spore air concentrations. However, the net effect of rainfall on spore emissions is unclear given that rainfall may act as a water source for the active emission mechanism and possibly aid spore maturation and germination. Preceding rainfall before peak emission may act to encourage spore emissions, whereas rainfall during peak emission may suppress spore air concentrations. Net radiation at the ground surface is known to represent the energy limit available to organic (as well as inorganic) systems (Oke 1987). Solar radiation is the driving force behind meteorological processes in the ABL, which in turn influence spore emissions. Net radiation is generally well correlated with net solar radiation during daylight hours (Oke 1987).

The main objective of our work is to infer the relationship of fungal spore emissions as a function of meteorological variables.

## Materials and methods

### Processing and analysis of fungal spore emissions

**Fig. 1:**
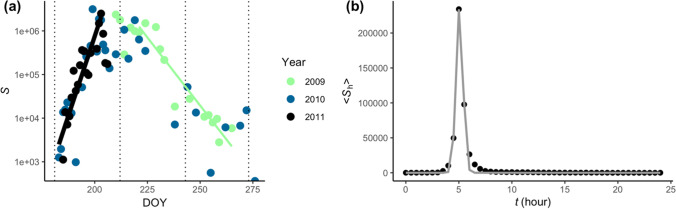
*H. fraxineus* near-ground atmospheric spore counts from Hietala et al. (2013) for (**a**) the growing season denoted *S* (number of spores day^−1^) for the years 2009 to 2011 and (**b**) the diurnal variations (number of spores h^−1^) averaged across all days and years, denoted by ⟨*S*_h_⟩, marked with black dots. Before averaging, we temporally shifted each diurnal dataset so that each daily maximum emission coincided with the average maximum emission time. The increase and decrease of ln(*S*) were fitted to linear models (solid black and green lines, respectively, in (**a**)). A Gaussian model was fitted to ⟨*S*_h_⟩, marked by the grey line in (**b**). Temporally shifting the data prior to averaging allowed the Gaussian to reveal the average shape of the emission peak. See Appendix B and C in Supplementary Material for further model details, parameter values and summary statistics. The observations made in 2009 only captured the decrease in the seasonal spore emissions, whilst in 2011 the measurements only captured the increase in the seasonal emissions

We analysed data, provided by Timmermann et al. (2011) and Hietala et al. (2013), of *H. fraxineus* spore counts recorded at 30-min intervals throughout the months of July to September for the years 2009 to 2011 (Fig. 1a). These data are derived from near-ground measurements of *H. fraxineus* atmospheric spore concentrations in a diseased ash stand near Ås, 30-km south of Oslo, Norway (59° 40′ 44′′ N, 10° 46′ 31′′ E).

We removed days with missing or corrupted data within the peak diurnal emission period (see Appendix A in Supplementary Material).

By maximum likelihood estimation (and a number of simplifications), it is possible to construct a simple analytical Gaussian model for the average diurnal variation of spore emissions which vary through the season:

1 $${{S}^{^{\prime}}}_{h}(d,h)=\frac{{S}^{^{\prime}}(d)}{I} {e}^{-{(h-\mu )}^{2}/(2{\sigma }^{2})}$$

where ${S}^{^{\prime}}(d)$ is the analytical model for the total daily emissions occurring on day-of-year *d* (see Fig. 1a for a graphical representation of *S’*), *I* is a constant and *h* is the hour of the day. The model parameters *μ* and *σ* are the average time of the peak spore emission and the average standard variation of *μ*, with values of 5.1 h and 0.33 h (Fig. 1b), respectively. See Appendix B and C in Supplementary Material for full details of (1), the model parameters and summary statistics. The simple analytical model (1) could be used as a basic (and numerically inexpensive) representation of spore emissions in an atmospheric dispersion model. However, it does not account for the variation about the mean changes and cannot adapt to changing environmental conditions.

A large amount of variability is apparent in the spore data of Hietala et al. (2013) (see Fig. 1a, Appendix B in Supplementary Material, or Fig. 3). Some of this variability is due to variability in the magnitude and timing of the daily emissions. Changing the timing of the spore emission peak by several hours has the potential to significantly alter the area of the resulting spore deposition (e.g. Savage et al. 2010). Spore emission timing was estimated by finding the time of maximum observed emission, denoted *t*_*peak*_, in each daily cycle and the spore emission magnitude was estimated by calculating the observed total daily spore emissions (*S*).

Careful analysis of the raw spore data revealed 7 days of ill-defined emission peaks (see Appendix A in Supplementary Material), which is relevant when considering the time of maximum emission. These days generally correspond to the start/end of the emission period, when total spore emissions are low. When considering *t*_*peak*_, these 7 days were removed from the analysis.

### Meteorological data

We downloaded publicly available, hourly meteorological data, provided by the Norwegian Institute of Bioeconomy Research (NIBIO 2019) in collaboration with the Norwegian Meteorological Institute (Table 1). The meteorological data are from two sites: Åsbakken (59º 40′ 7′′ N, 10° 46′ 8′′ E) and Ås (59° 39′ 38′′ N, 10° 46′ 55′′ E). The Åsbakken data was used whenever possible, since this site was closest to the spore observation site (approximately 400 m) and also in an orchard. The Ås site, located in an open field less than 2 km from the spore observation site, was used for all variables that were not available from Åsbakken (Table 1). Meteorological variables that were measured from both stations show a high correlation (e.g. the Spearman rank correlation coefficients for *U*, *T*_*a*_ and *R* are 0.96, 0.99 and 0.81, respectively). Accurate soil moisture data was missing for 2011 and part of 2010. We therefore removed soil moisture from our analysis in order to maximise the number of observations. The final number of observations used to model both *t*_*peak*_ and *S* was 61 (15 from 2009, 27 from 2010 and 19 from 2011).

**Table 1:** The 10 meteorological variables, their three averaging windows and their use in the models for *t*_peak_ and log_10_(*S*). Squares marked - are variables that were excluded a priori based on the physics of the system*.* Variables used in the model for time of maximum spore emission are indicated with a *t*_peak_. Variables used in the model for total daily spore emissions are indicated with log_10_(*S*). Squares marked X indicate variables that were excluded due to collinearity

			Averaging window length		
Variable name & symbol	Units	Measurement details	window 1 (± 2 h)	window 2 (1 day)	window 3 (5 days)
Rainfall, *R*	mm h^−1^	RR, (1), ± 0.1 mm/h	*t*_peak_, log_10_(*S*)	X log_10_(S)	*t*_peak_ X
Soil moisture, $\theta$	1	VAN1, (2), first 10 cm, Volumetric, ± 0.02	-	-	-
Leaf moisture, *M*_*s*_	min/h	BTff, (1), ∼ 2-m a.g.l., accuracy unknown	*t*_peak_, log_10_(*S*)	*t*_peak_ X	X log_10_(*S*)
Relative humidity, *U*	1	UMf, (1), hourly mean, ± 2% (0 to 90%)	-	-	-
Soil temperature, *T*_*g*_	^o^C	TJM1, (2), 1-cm deep, hourly mean, ± 0.2 K	X	*t*_peak_, log_10_(*S*)	X
Surface temperature, *T*_*s*_	^o^C	TS, (2), hourly mean, ± 0.2 K	-	-	-
Air temperature, *T*_*a*_	^o^C	TMf, (1), 2-m a.g.l., hourly mean, ± 0.2 K	-	-	-
Net radiation, *R*_*n*_	W m − ^2^	RN, (2), surface flux, hourly mean, < 10% of each days integrated radiation	*t*_peak_, log_10_(*S*)	*t*_peak_, log_10_(*S*)	*t*_peak_, log_10_(*S*)
Wind speed, *|*u*|*	m s^−1^	FM2, (2), 2-m a.g.l., hourly mean, horizontal speed, 1% ± 0.1 m/s	*t*_peak_, log_10_(*S*)	*t*_peak_, log_10_(*S*)	-
Frost, *F*	Presence/absence		-	*t*_peak_, log_10_(*S*)	-

Notes: Acronyms within ‘Measurement details’ are the NIBIO variable names. Locations of observations are specified using the numbers in brackets, where 1 = Åsbakken and 2 = Ås, and equipment error estimates are provided. We derived an additional variable for the presence of frost when surface temperature is less than 0 °C and leaf moisture is non-zero. Leaf moisture was observed using a leaf wetness sensor, which gives the time in minutes the leaf is wet within each hour (see Campbell Scientific 2020 for more information)

From a physical understanding of the system (see Oke 1987, Chapters 1 and 4), three pairs of variables (*T*_*s*_ and *R*_*n*_, *T*_*s*_ and *T*_*a*_, *M*_*s*_ and *U*) are expected to be highly correlated. We therefore excluded *T*_*s*_, *T*_*a*_ and *U* from our analysis a priori (Table 1). Our a priori expectation was supported by observed strong correlations in the hourly data between: *T*_*s*_ and *R*_*n*_ (Spearman rank coefficient 0.77), *T*_*s*_ and *T*_*a*_ (Spearman rank coefficient 0.91) and *M*_*s*_ and *U* (Spearman rank coefficient 0.72).

The six remaining meteorological variables were averaged across three time windows: 2 h either side of *t*_*peak*_ (window 1), 2–24 h before *t*_*peak*_ (window 2) and less than 5 days before *t*_*peak*_ (window 3). These three windows capture immediate (window 1), near-term (window 2) and longer-term (window 3) meteorological conditions (see Introduction). Window 1 was chosen to encompass the mean emission peak (guided by *σ* in the Processing and analysis of fungal spore emissions section). We excluded the 5-day average wind speed (window 3) a priori since it is unlikely to impact spore emissions. Outside of the winter months, frost typically occurs during the early morning hours before sunrise given enough moisture and a freezing surface. However frost may also occur earlier in the night (Oke, 1987). The window 1 frost average was dropped due to the rarity of frost occurrence. A window 3 average of frost is likely hard to interpret and so was removed from the analysis. A further six averaged variables were also excluded due to collinearity (see Table 1 and the “Statistical analysis” section). This gave a final set of 10 continuous meteorological variables and one binary variable (frost, window 2).

### Statistical analysis

We constructed two generalised additive models (GAMs, see Wood 2017): one had the time at maximum *H. fraxineus* spore release, *t*_peak_, as a response variable, and the other had the log-transformed daily spore emission, log_10_(*S*), as a response variable. All models had the factor frost (window 2) as a parametric term and 10 smoothed meteorological variables. Variables for these smoothed terms were selected by taking the variable with the greatest Spearman rank correlation with the response variable, removing all other collinear variables (defined here as having a Spearman rank correlation greater than 0.6) and then repeating until all 14 variables had been used. The final selection of variables for the two models is given in Table 1.

All the smoothed terms used a thin plate spline basis with a basis dimension of three. Variable selection was performed by imposing an additional penalty on functions in the smoothing penalty null-space (Marra and Wood 2011). No interactions were included because the data were too sparse and data exploration showed no pattern of interactions.

We fitted models to a random 80% of the data from years 2010 and 2011 (a total of 22 and 16 observations from 2010 and 2011, respectively) and validated the model using the remaining 20% of the 2010 and 2011 data. Data from 2009 was used as independent validation data (see below). We performed model fitting and validation on 1000 different randomised 80%:20% data partitions; therefore we have combined a Monte-Carlo approach with GAMs. Models were fitted using generalised cross validation in the mgcv package in R, version 3.5.2 (R Core Team 2018; Wood 2011). A model’s effective degrees of freedom were multiplied by 1.4 to increase the amount of smoothing and to avoid overfitting (Wood 2017). Residuals were assumed to follow a normal distribution and to have no temporal autocorrelation. The model assumptions were tested and confirmed for 10 of the 1000 fitted models.

We quantified the importance of each smoothed term in a model by calculating the number of times (from the 1000 randomised fits) a term had been retained in a model (i.e. had an effective degrees of freedom greater than 10^−4^). Smoothed terms that were retained in more than 70% of all selected models were highlighted. We also reported the fitted relationship for the most commonly selected model (the terms in the most commonly selected model may not be the same as terms that were retained in more than 70% of all models). Model fit was estimated from the *R*^2^ of both fitted and validation models. We visualised the fitted relationships for a variable using a contour plot of the relative density of predictions across all 1000 randomised fits. We calculated the predicted relationship from each randomised fit by varying the variable across its observed range whilst setting all other variables to their observed median value. We also averaged the fitted coefficients across all 1000 models by calculating the median coefficient (and standard error) for each smooth term.

Further model validation was performed by predicting log_10_(*S*) and *t*_peak_ for 2009. All 1000 models for each response were used to make predictions for 2009. These models were then averaged by taking the median point prediction and 95% confidence interval. The Pearson correlation coefficient between predictions and observations of log_10_(*S*) and *t*_peak_ was used as a measure of model predictive performance.

The effect of omitting soil moisture was investigated by repeating our analysis using the dataset where soil moisture was available (2009 and part of 2010). The results of this are presented in Appendix F (Supplementary Materials).

Finally, we repeat our analysis using a random forest approach (Liaw and Wiener 2002), allowing comparison with our GAM method. The application of the random forest mirrors the original GAM approach in using 1000 regression trees to build the random forest, using the same size for the out-of-bag sample and keeping data from 2009 as an independent validation dataset. The random forest analysis is presented in Appendix G in Supplementary Materials and summarised below.

## Results

### Model fit and predictive performance

The model fit and predictive ability (i.e. validation *R*^2^) of our models for total daily spore emissions, log_10_(*S*), and the timing of peak spore emission, *t*_peak_, depended upon how the data were partitioned into fitting and validation datasets (Figure D1, Appendix D in Supplementary Material). Half of the 1000 models for both log_10_(*S*) and *t*_peak_ had a validation *R*^2^ greater than 42% and 61%, respectively (Figure D1). There is a negative correlation between *R*^2^ from the fitting data and the *R*^2^ from the validation data (i.e. predictive ability) due to the bias-variance trade off (i.e. high fitted *R*^2^ tend to correspond with model over-fitting). After model selection, the complexity of the models for both log_10_(*S*) and *t*_peak_ ranged from three to nine smoothed terms, but most models had five to seven terms (Table 2). On average, models for log_10_(*S*) with intermediate complexity (six to seven terms) had the greatest predictive power, whereas models for *t*_peak_ with the greatest complexity (nine terms) had the greatest predictive power (Table 2). Including soil moisture in the analysis did not increase the predictive power of the models (Table F2, Appendix F) but did result in selected models with fewer terms and a clear decline in predictive performance for selected models with a large number of terms (more than five terms).

**Table 2:** The frequency (across 1000 fitted models to random data partitions) with which different model complexities (i.e. number of terms) were selected and their median *R*^2^ for the validation data

Number of smooth terms	3	4	5	6	7	8	9
log_10_(S) model							
Frequency	2	13	84	344	408	131	18
Median validation *R*^2^	0.16	0.32	0.37	0.46	0.43	0.42	0.40
*t*_*peak*_ model							
Frequency	17	143	372	303	121	41	3
Median validation *R*^2^	0.49	0.57	0.58	0.65	0.76	0.85	0.85

### Total daily emission of spores

Across all 1000 models, 112 unique combinations of smooth terms were selected. The most common combination of smooth terms (selected for 184 of the 1000 models) contained the weekly average net radiation and leaf moisture (window 3), daily average soil temperature and rainfall (window 2) and immediate net radiation and leaf moisture (window 1). Across all 1000 models, the same terms that appeared in the common model (net radiation, rainfall, leaf moisture and soil temperature) were selected in over 70% of all models (Table 3). Longer-term (window 3) variables are important predictors. Both window 3 variables (net radiation and leaf moisture) were present in at least 89% of all models. However, near-term (window 2) and immediate (window 1) variables are also important. Net radiation (window 1) and soil temperature (window 2) were present in 95% and 96% of all models.

**Table 3:** The variables and timescale for the 10 smooth terms in the GAM model for log_10_(*S*) (Table 1) and the proportion of the 1000 randomisations for which each term was selected in the final model. Terms selected in more than 70% of models are highlighted in bold

Variable	Timescale	Selection proportion
**Net radiation**	**Window 3**	**1.00**
**Soil temperature**	**Window 2**	**0.96**
**Net radiation**	**Window 1**	**0.95**
**Leaf moisture**	**Window 1**	**0.92**
**Leaf moisture**	**Window 3**	**0.89**
**Rainfall**	**Window 2**	**0.76**
Net radiation	Window 2	0.49
Wind speed	Window 1	0.36
Rainfall	Window 1	0.18
Wind speed	Window 2	0.11

**Fig. 2:**
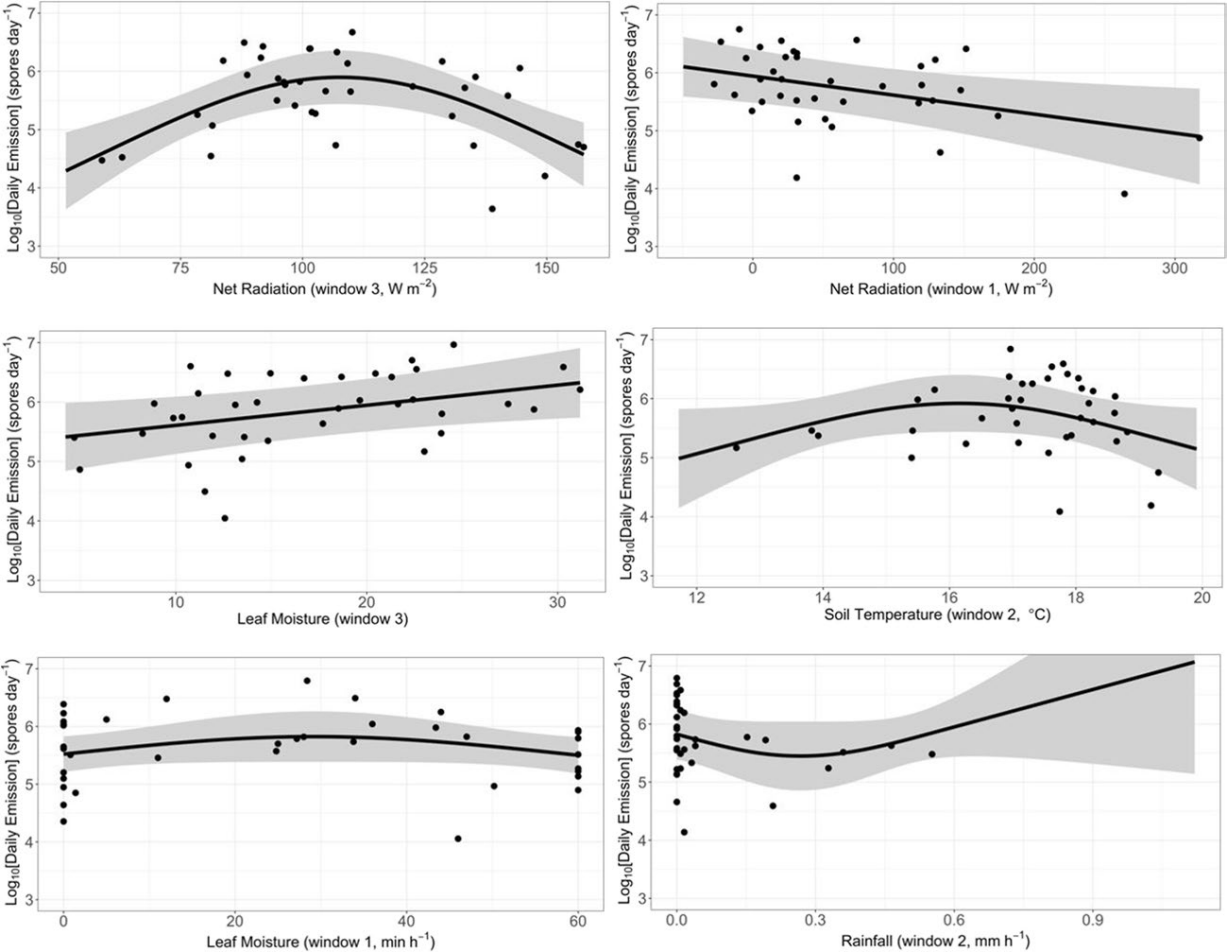
The marginal predictions (black line) and 95% confidence intervals (grey region) from the most common model for total daily spore emissions (six smoothed terms). Residuals are shown by solid circles. Predictions are calculated at the median values for the remaining three variables

The marginal model predictions from this common model show that daily spore emissions peak at intermediate values of weekly average net radiation (window 3) and daily average soil temperature (window 2) and increase linearly with weekly average leaf moisture (window 3) and decrease linearly with immediate net radiation (window 1, Fig. 2). The relationship with rainfall (window 2) and immediate leaf moisture (window 1) appears weak. These relationships appear relatively robust. The relative density of model predictions, averaged over all 1000 models, gives similar qualitative relationships to the predictions of the commonest model (Table 4, Figure E1, Appendix E in Supplementary Material).

**Table 4:** The fitted coefficients and their standard errors for total daily spore emissions, log_10_(*S*). Values are the medians across all 1000 fitted models. Terms in bold are selected in over 70% of all models

Smooth term	Coefficient	Median estimate	Median standard error
**Intercept**	**-**	**5.4**	**0.17**
**Net radiation**	**Coefficient 1**	**1.4**	**0.36**
**(Window 3)**	**Coefficient 2**	** − 2.7 × 10**^**–7**^	**2.8 × 10**^**−4**^
**Net radiation**	**Coefficient 1**	**2.2 × 10**^**−7**^	**6.7 × 10**^**−4**^
**(Window 1)**	**Coefficient 2**	** − 0.24**	**0.096**
**Leaf moisture**	**Coefficient 1**	**9.3 × 10**^**−7**^	**9.2 × 10**^**−4**^
**(Window 3)**	**Coefficient 2**	**0.28**	**0.097**
**Soil temp**	**Coefficient 1**	**0.83**	**0.37**
**(Window 2)**	**Coefficient 2**	**2.8 × 10**^**−8**^	**1.7 × 10**^**−4**^
**Leaf moisture**	**Coefficient 1**	**0.55**	**0.33**
**(Window 1)**	**Coefficient 2**	** − 1.3 × 10**^**−7**^	**1.7 × 10**^**−4**^
**Rainfall**	**Coefficient 1**	** − 0.36**	**0.41**
**(Window 2)**	**Coefficient 2**	** − 2.8 × 10**^**−7**^	**2.1 × 10**^**−4**^
Rainfall	Coefficient 1	3.5 × 10^**−**7^	1.4 × 10^**−**3^
(Window 1)	Coefficient 2	3.3 × 10^**−**7^	2.1 × 10^**−**4^
Net Radiation	Coefficient 1	3.0 × 10^**−**7^	6.3 × 10^**−**4^
(Window 2)	Coefficient 2	9.0 × 10^**−**7^	3.2 × 10^**−**4^
Wind speed	Coefficient 1	− 2.2 × 10^**−**6^	9.5 × 10^**−**4^
(Window 1)	Coefficient 2	− 7.5 × 10^**−**9^	1.4 × 10^**−**4^
Wind speed	Coefficient 1	− 9.5 × 10^**−**8^	6.1 × 10^**−**4^
(Window 2)	Coefficient 2	− 7.1 × 10^**−**8^	1.6 × 10^**−**4^

**Fig. 3:**
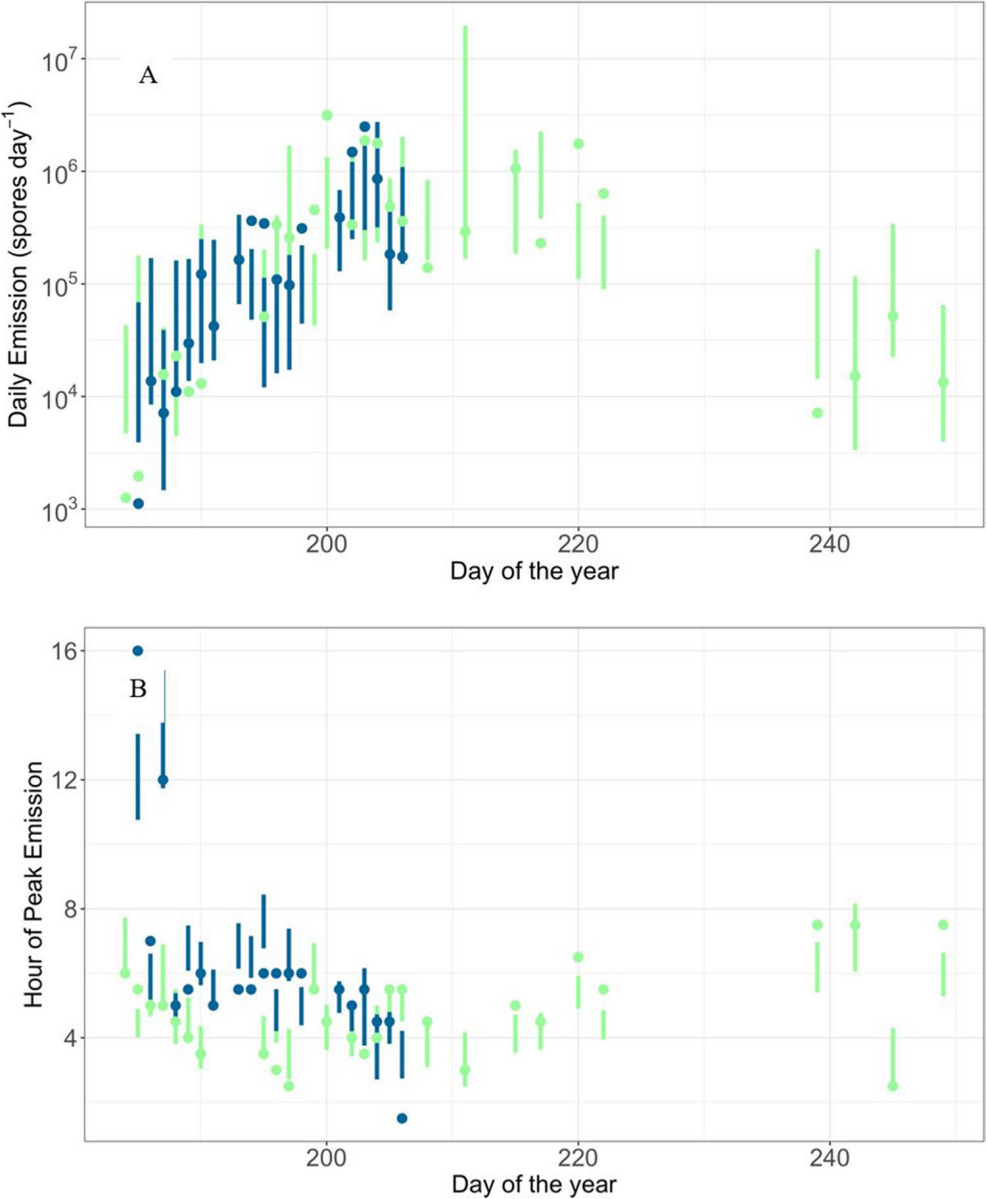
The observed daily spore emission (**A**, circles) and hour of peak emission (**B**, circles) for 2010 (green) and 2011 (blue). The vertical lines show the predicted 95% confidence intervals from the most common model

The model prediction using the most common model for the total daily spore counts is compared against the observations in Fig. 3A. The model is shown to generally capture the trends in the observations well and most observations fall within the 95% confidence intervals for the prediction.

**Fig. 4:**
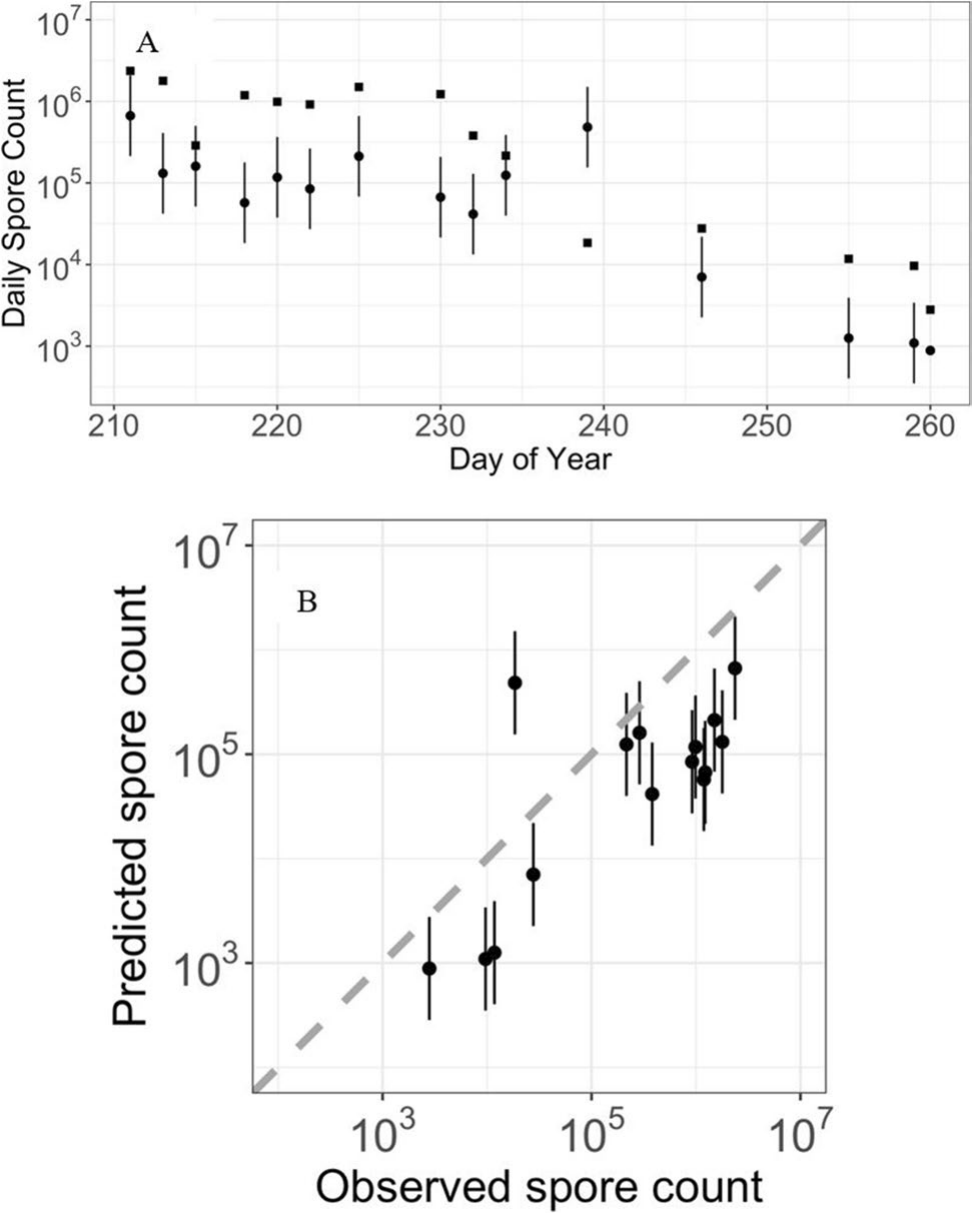
**A** The observed daily spore counts for 2009 (squares) as a function of time and the model predictions (circles). **B** Observed daily spore counts for 2009 versus predictions (*R*^2^ = 58%). Dashed diagonal line represents observed equal to predicted. Bars represent 95% confidence intervals. Predictions and confidence intervals are medians across all 1000 fitted models

Data for 2009 can be used as an independent test of the fitted models. The median prediction from the 1000 models of log_10_(*S*) for 2009 tends to underpredict (Fig. 4). However, the predictions do capture the relative changes in spore counts (*R*^2^ = 58%), suggesting that the commonly selected variables have some predictive ability for changes in daily spore counts.

Including soil moisture as a possible term in the model reduced the importance of soil temperature, net radiation (window 1) and leaf moisture (window 1), but the importance of net radiation (window 3), leaf moisture (window 3) and to a lesser extent rainfall (window 2) were maintained (see Table F3 and Figure F1, Appendix F). Soil moisture did not emerge as an important variable and was selected in only 11% of models.

#### Timing of daily peak emissions

**Fig. 5:**
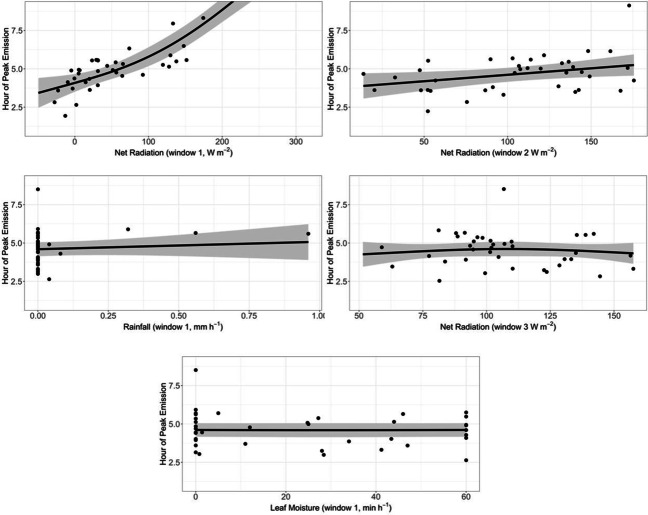
The marginal predictions (black line) and 95% confidence intervals (grey region) from the most common model of timing of peak spore emissions (five smoothed terms). Residuals are shown by solid circles. Predictions are calculated at the median values for the remaining five variables

Model term selection gave 143 unique combinations of smoothed terms from the 1000 models for the timing of the daily peak in spore emission. The most common model had five smooth terms (net radiation windows 1, 2 and 3, leaf moisture window 1 and rainfall window 1). This common model occurred 88 times out of 1000 (Fig. 5), with the second most common model occurring 67 times. Across all 1000 models four variables (net radiation for windows 1, 2 and 3, and rainfall for window 1) were selected in over 70% of all models (Table 5). Immediate net radiation (window 1) clearly has the strongest association with the timing of peak spore counts. However, near term and longer-term net radiation (windows 2 and 3) are also associated with the timing of the peak.

**Table 5:** The variables and timescale for the 10 smooth terms in the GAM model for *t*_peak_ (Table 1) and the proportion of the 1000 randomisations for which each term was selected in the final model. Terms selected in more than 70% of models are highlighted in bold

Variable	Timescale	Selection proportion
**Net radiation**	**Window 1**	**1.00**
**Rainfall**	**Window 1**	**0.88**
**Net radiation**	**Window 2**	**0.86**
**Net radiation**	**Window 3**	**0.72**
Leaf moisture	Window 1	0.60
Soil temperature	Window 2	0.47
Net radiation	Window 3	0.46
Leaf moisture	Window 2	0.44
Wind speed	Window 2	0.34
Rainfall	Window 3	0.16
Wind speed	Window 1	0.03

Across all 1000 models, the strongest consistent relationship is for net radiation across all time-scales, but with net radiation near the time of peak emission (window 1) having the strongest effect. Increased immediate net radiation (window 1) is associated with a delay in the timing of the daily peak in spore emissions (Table 6, Figure E2, Appendix E in Supplementary Material). The relationship with rainfall (window 1) is included in over 70% of models, but the effect size is weak (Fig. 5). Similarly leaf moisture (window 1) is included in the most commonly selected model but with an effect size that is small compared to the uncertainty (Table 6, Fig. 5).

**Table 6:** The fitted coefficients and their standard errors for the timing of peak daily spore emissions, *t*_peak_. Values are the medians across all 1000 fitted models. Terms in bold are selected in over 70% of all models

Smooth term	Coefficient	Median estimate	Median standard error
**Net radiation**	**Coefficient 1**	**1.9**	**0.75**
**(Window 1)**	**Coefficient 2**	**2.1**	**0.19**
**Net radiation**	**Coefficient 1**	**1.0**	**1.5 × 10**^**−3**^
**(Window 2)**	**Coefficient 2**	**0.37**	**0.16**
**Rainfall**	**Coefficient 1**	**1.4 × 10**^**−5**^	**5.9 × 10**^**−3**^
**(Window 1)**	**Coefficient 2**	**0.13**	**0.11**
**Net radiation**	**Coefficient 1**	− **2.3 × 10**^**−6**^	**0.097**
**(Window 3)**	**Coefficient 2**	**6.8 × 10**^**−9**^	**7.5 × 10**^**−4**^
Leaf moisture	Coefficient 1	3.3	1.4 × 10^**−**3^
(Window 2)	Coefficient 2	− 5.4 × 10^**−**6^	1.2 × 10^**−**3^
Leaf moisture	Coefficient 1	− 0.13	0.26
(Window 1)	Coefficient 2	− 3.7 × 10^**−**7^	3.7 × 10^**−**4^
Wind speed	Coefficient 1	− 8.7 × 10^**−**7^	1.6 × 10^**−**3^
(Window 2)	Coefficient 2	− 1.7 × 10^**−**6^	6.4 × 10^**−**4^
Rainfall	Coefficient 1	− 2.3 × 10^**−**6^	2.0 × 10^**−**3^
(Window 3)	Coefficient 2	6.8 × 10^**−**9^	2.9 × 10^**−**4^
Soil temp	Coefficient 1	− 3.3 × 10^**−**5^	5.1 × 10^**−**3^
(Window 2)	Coefficient 2	− 1.9 × 10^**−**7^	3.2 × 10^**−**4^
Wind speed	Coefficient 1	− 8.0 × 10^**−**8^	1.4 × 10^**−**3^
(Window 2)	Coefficient 2	− 1.0 × 10^**−**7^	3.0 × 10^**−**4^

The model prediction using the most common model for the hour of peak emission is compared against the observations in Fig. 3B. The model is shown (Fig. 3B) to generally capture the trends in the observations with most observations falling within the 95% confidence intervals for the prediction (Fig. 4).

**Fig. 6:**
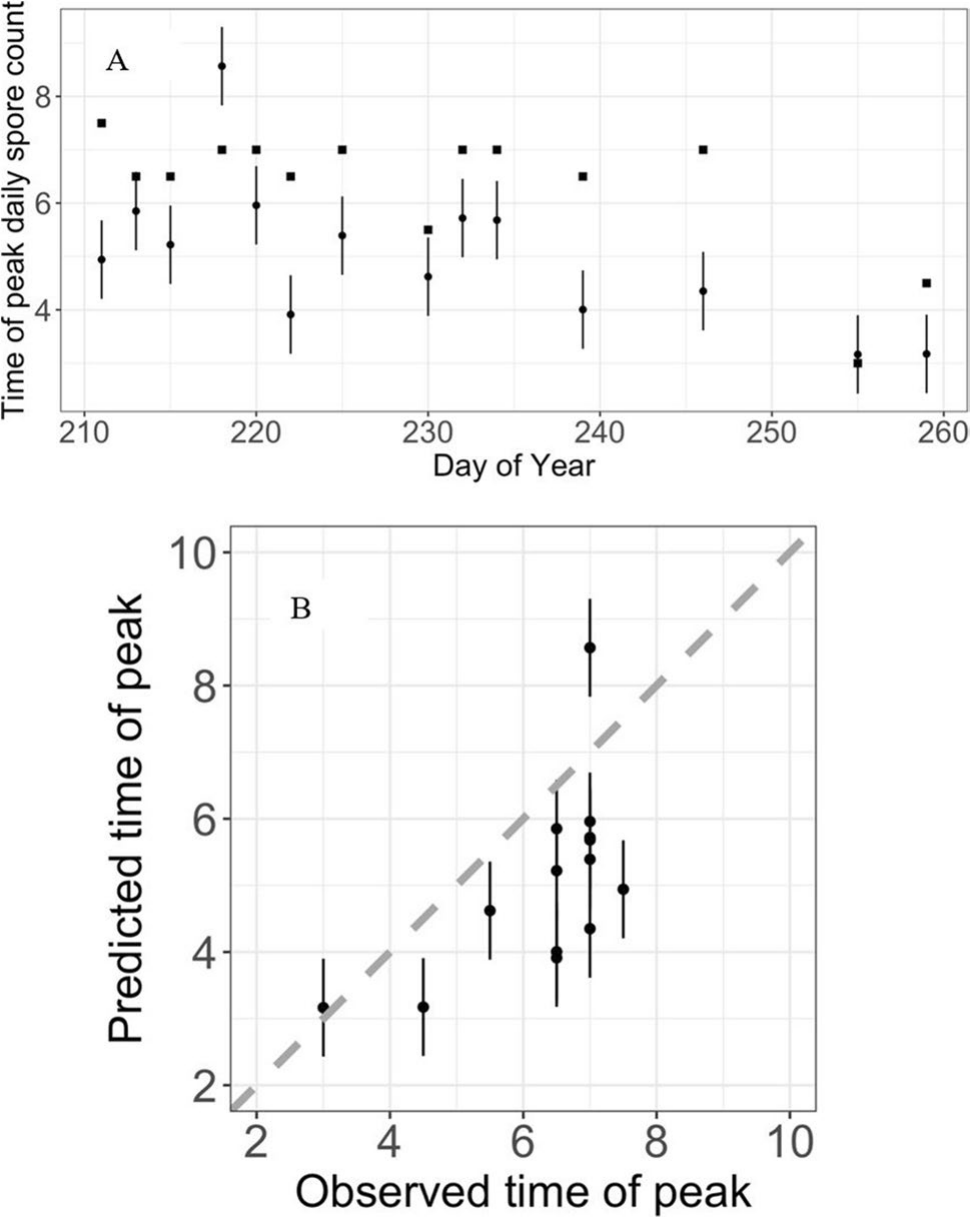
**A** The observed timing (hours since 00:00 h) of peak spore counts for 2009 (squares) as a function of time and the predictions (circles). **B** Observed timing of peak spore counts for 2009 versus predictions (*R*^2^ = 37%). Dashed diagonal line represents observed equal to predicted. Bars represent 95% confidence intervals. Predictions and confidence intervals are medians across all 1000 fitted models

Validation using data from 2009 shows a tendency to underpredict the timing of the peak spore count (Fig. 6). However, there is some predictive skill in the relative changes in the timing of the peak from day to day (*R*^2^ = 37%, Fig. 6B).

Including soil moisture as a possible term in the model showed that soil moisture may be associated with the timing of the daily peak in spore emissions (Table F4, Appendix F). A soil moisture (window 1) term and a net radiation (window 1) term were selected in all 1000 models. These models predict that increasing soil moisture and increasing net radiation delay the time of peak spore emissions.

#### Random forest approach

We repeated our analysis using a random forest approach and found that the random forest gives poor overall predictive performance but does select broadly similar variables to the GAM method (see Appendix G in Supplementary Materials).

## Discussion

We have shown that the quantity of *H. fraxineus* spores emitted per day and the daily timing of the peak in spores can be associated with weather conditions both at the time of spore emission and over the hours and days preceding the emission of spores.

Several factors make it likely that our models are missing key phenological variables, indicating that further work is required before the models can be used operationally. The results that support this are (1) the general under-prediction of total daily spore counts and peak emission timing when predicting spore emissions in a different year to that used to fit the model, (2) the near-exponential seasonal increase and decrease of average total daily spore counts and (3) the consistent association of total daily emissions with 5-day average net radiation.

In the “Processing and analysis of fungal spore emissions” section, the increase and decrease of seasonal spore emissions were found to be nearly exponential, indicating a population growth that is not limited by resources. The models assessed by Eikemo et al. (2010) for estimation of seasonal ascospore increase of the ascomycete *Venturia pyrina* also have an essentially exponential form. However, there is clear day-to-day variation in the observed spore emissions of *H. fraxineus* ascospores, which must be accounted for in order to accurately predict spore dispersion and infection (e.g. Savage et al. 2010).

It is possible that our 5-day average net radiation is partly acting as a proxy for the main seasonal peak of spore emissions (and other possibly more fundamental phenological factors). Net radiation averaged over 5 days preceding spore release (i.e. the longer-term timescale) shows a particularly consistent association with the total daily emissions of *H. fraxineus* spores (Fig. 2). This longer-timescale average of net radiation reveals the generally diminishing solar insolation from late summer to autumn (not shown). Average net radiation is the primary variable where the relationship with spore emissions peaks at intermediate values (5-day average net radiation of 80 to 120 W m^−2^). This peak reflects the seasonal trend in spore emissions because 5-day average net radiation decreases roughly linearly from July through September (not shown), whilst generally there is a peak in the spore emissions close to half-way through this observation window (Fig. 1a). It appears that our 5-day average net radiation is accounting for some of the near-weekly changes as well as the seasonal change.

Other important phenological factors might include weather conditions during the winter and spring preceding the annual peak in spore emissions. The study of Hietala et al. (2018) analysed *H. fraxineus* spore emissions observed near Bergen from 2011 to 2017 and found a significant correlation between annual maximum spore levels and cumulative ‘degree days’. In other words, it appears that the fungus is more able to reproduce during generally warmer years. Average net radiation can be linked to a ‘degree day’ diagnostic, which is essentially an integral over time of surface temperature, and surface temperature may be written as a function of surface radiation variables (Oke, 1987). Hietala et al. (2013) found a rapid increase in the spore emissions (of *H. fraxineus*) in mid-July when the cumulative growing degree days had reached 600. The models assessed by Eikemo et al. (2010), and the model used by Stensvand et al. (2005) used degree days to model the smooth mean seasonal changes neglecting shorter timescale natural variations. However, Hietala et al. (2018) also found an inter-annual exponential growth in the maximum seasonal spore number between 2012 and 2015. This was attributed to the fungus moving to its new environment near Bergen and initially having unrestricted access to resources. Therefore, although it does seem likely that accounting for weather conditions prior to the annual emission peak will improve the inter-annual predictive skill of our models, it is also possible that further factors (e.g. availability of host resources) would be required.

Differences in annual weather conditions across Europe (i.e. climatic variations) may also be an important factor in the variation of the onset of the annual peak emission across Europe. The onset of fruit body formation and hence sporulation of *H. fraxineus* differs on the order of several months across Europe (Hietala et al. 2013, 2018; Kirisits & Cech 2009; Grosdidier et al. 2018; Chandelier et al. 2014; Dvorak et al. 2016). The performance of our model over an inter-annual basis and when applied to other European locations should be the topic of future work, because our current results are based upon data from only three years of spore observations, from one location. Furthermore, our methods could be applied to other airborne fungal pathogens with similar dispersal mechanisms to test the model’s performance.

Despite the drawbacks noted above, our models retain predictive skill both when predicting emissions in years used for the model fit, as well as for the independent 2009 dataset.

Our results showed that increased net radiation during the peak emission period (i.e. window 1) causes a reduction in the total daily spore emissions, modulating the effects of longer-term trends in net radiation.

We found that the quantity of *H. fraxineus* spores emitted per day is affected more by leaf (surface) moisture than rainfall. We found that spore emissions increase with 5-day average leaf moisture (see Fig. 2), which corresponds well with the theory that humid air aids spore maturation and germination, as discussed by Timmermann et al. (2011) and Hietala et al. (2013). Moisture is also known to be important for *H. fraxineus*’ active emission mechanism (Ingold, 1999). Interestingly, the seasonal change of longer-term leaf moisture (window 3) generally follows the seasonal change in daily spore emissions (not shown). We have confirmed the importance of moisture for the quantity of spore emissions and isolated the types of moisture and ranges of moisture values that the fungus is most sensitive to for our dataset. Moisture is also key for ascospore production of other ascomycetes, such as *Venturia inaequalis* (Stensvand et al. 2005, 2009).

The strongest effect sizes associated with the timing of spore release are for net radiation close to the time of release, with increased net radiation delaying the time of the peak emission. The observed time of peak spore emissions varies by several hours, which is likely to cause strong variation in the atmospheric dispersion and deposition of spores (e.g. Savage et al. 2010).

It was surprising that both the daily quantity and timing of spore release were not particularly sensitive to wind speed or rainfall, since both are well known to strongly affect aerosols (Di-Giovanni et al. 1995; Aylor 2003). However, it should be noted that the meteorological observations were not optimised for correlating weather variables with spore emissions and the weather observing stations were at least 400 m away from the spore observation site. In particular, it is likely that wind conditions varied between the weather observing stations, located on open ground and in an orchard, to the conditions within the woodland ash stand. However, for the variables other than wind speed, we found good correlation between the two weather stations, despite their separation of about 1 km. Soil moisture observations were also limiting, forcing us to remove this variable from the main analysis. However, there is a suggestion that soil moisture is potentially an important variable for the timing of the daily peak in spore emissions. Future work should consider more bespoke observation campaigns combining weather and spore observations.

In the study of Hietala et al. (2018) relationships were analysed (using Spearman rank correlation coefficients) between spore emissions summed from midnight to noon and daily averages of temperature, precipitation, relative humidity and wind speed. With a few exceptions, no significant correlations were found. However, it should be noted that the weather station was located approximately 7 km away from the spore observation site. This may well indicate that *H. fraxineus* is most sensitive to its immediate environment rather than mesoscale meteorology. This comparison also suggests that it is likely the combination of different weather factors that influence *H. fraxineus* spore emissions rather than any one single variable.

With only approximately 60 data points underlying the statistical analysis, we have been careful to remove meteorological variables with a priori reasons not to be associated with *H. fraxineus* spore release. We have also limited the complexity of the statistical model by not including interaction terms and keeping the smoothing relatively strong so that we only capture broad non-linearity in the associations. Despite our relatively small dataset, we have still randomly held out 20% of the data from the years used for the model fitting (2010 and 2011) as well as keeping the 2009 data as an independent validation dataset. Although this reduces the information available to the model fitting, it does allow us to apply three methods for validating the fitted models. Firstly we can estimate the predictive performance of a fitted model on the 20% of the data held out from the fitting process, showing reasonable predictive ability (*R*^2^ ~ 0.5). Secondly, we can use predictions for 2009 as a second measure of predictive performance. This shows an inter-annual predictive ability (*R*^2^ ~ 0.5) comparable to the intra-annual predictions. Thirdly, by repeatedly fitting our model to different randomised data, we assess the variability in model selection and the relative degree to which our models are over- or under-fitting the data. This can be seen in the negative association between *R*^2^ from the fitted dataset and *R*^2^ from the validation dataset (Figure D1 in Supplementary Material).

Our findings provide a methodology for estimating the magnitude and timing of spore emissions based on meteorological observations. Using a generalised additive model, framework allows the model flexibility to capture non-linear relationships but also the ability to control the smoothness of a relationship. In this GAM framework, a model for either response variable will have the general form:

$$g\left({u}_{i}\right)={f}_{1}\left({x}_{1i}\right)+ {f}_{2}\left({x}_{2i}\right)+{f}_{3}\left({x}_{3i}\right)+\dots + {\varepsilon }_{i}$$

where $g\left({u}_{i}\right)$ is some function of the response variable (simply the response variable itself for the timing of the peak spore emissions and the logarithm of the daily spore emissions). Note that the above equation neglects any parametric variables (the binary frost variable in our case) since we did not find it to be significant. The terms on the right side of the equation are smooth functions ($f$) of our covariates ($x$) and an error term (*ε*) that is assumed to follow a normal distribution. The GAM framework also gives the ability to perform model selection of terms. For this we used a smoothing penalty in the null-space (Marra & Wood, 2011), but other approaches to model selection in GAMs exist (Wood 2017).

## Conclusions

Our results indicate that the magnitude and timing of *H. fraxineus* spore emissions are strongly affected by the meteorological and associated land-surface conditions in the close vicinity of the pathogen. A novel method combining a Monte-Carlo approach with GAMs has quantified the uncertainty in the structure of the model and the relative degree to which our models are over- or under-fitting the data. We find that the magnitude of spore emissions is associated with trends in weather variables during the emissions (net radiation) as well as in the preceding day (soil temperature) and week (net radiation and leaf moisture) prior to the emissions. Average net radiation and soil temperature are the only variables where the number of spores peak at intermediate values. Increases in 5-day average leaf moisture are associated with increased daily spore emissions, and the immediate net radiation modulates the effect of the longer-timescale 5-day average net radiation. Increases in immediate timescale net radiation were found to delay the hour of peak spore emission. In line with previous work, we have confirmed the importance of moisture for the quantity of spore emissions. We found that the seasonal peak in spore emissions has a near-exponential increase and decrease, and the mean daily emission peak is approximately Gaussian, allowing the construction of a simple analytical model for the mean changes (neglecting model residuals). Our GAM framework and standard meteorological variables provide a more accurate method for estimating *H. fraxineus* spore emissions, accounting for variations about the means and adapting to changing environmental conditions.

Future work should consider a more be-spoke observation campaign with observations of fungal spores, meteorology and surface conditions at the same location. More work is needed to determine the generality of our results, in particular, inter-annual model performance, whether or not the models can be applied in other locations across Europe and beyond, and whether they are useful for similar fungal pathogens. Work is needed to develop a more mechanistic understanding of how environmental conditions (atmospheric, land surface, availability of host resources etc.) affect *H. fraxineus* spore emissions.

## Supplementary Information

Below is the link to the electronic supplementary material.

Supplementary file1 (DOCX 7234 KB)

## Data Availability

The datasets generated during and/or analysed during the current study are available in the Zenodo repository, [https://doi.org/10.5281/zenodo.4467823].

## Code availability

The R code used for the statistical analysis in the current study is available in the Zenodo repository, [https://doi.org/10.5281/zenodo.4467787].
