# Supplementary material for: Meteorological factors associated with the timing and abundance of Hymenoscyphus fraxineus spore release
Source: Int J Biometeorol. 2021 Nov 11;66(3):493–506. doi: 10.1007/s00484-021-02211-z (PMC8850239; doi:10.1007/s00484-021-02211-z)
Supplement: Supplementary file 1 — Supplementary file1 (DOCX 7234 KB) [file 484_2021_2211_MOESM1_ESM.docx]

**Supplementary Material:**

**Meteorological factors associated with the timing and abundance of *Hymenoscyphus fraxineus* spore release**

Paul Burns^1^, Volkmar Timmermann^2^, Jon M Yearsley^3*^

^1^College of Engineering, Mathematics and Physical Sciences, Harrison Building, Streatham Campus, University of Exeter, North Park Rd, Exeter, UK, EX4 4QF. [p.burns2@exeter.ac.uk](mailto:p.burns2@exeter.ac.uk) <https://orcid.org/0000-0001-7954-1173>

^2^ Division of Biotechnology and Plant Health, Norwegian Institute of Bioeconomy Research (NIBIO), P.O. Box. 115, 1431 Ås, Norway. [volkmar.timmermann@nibio.no](mailto:volkmar.timmermann@nibio.no)

^3^ University College Dublin, School of Biology & Environmental Science & UCD Earth Institute, Belfield, Dublin 4, Ireland. [Jon.Yearsley@ucd.ie](mailto:Jon.Yearsley@ucd.ie) <https://orcid.org/0000-0003-1838-0454>

* Corresponding author: Paul Burns p.burns2@exeter.ac.uk

**APPENDIX A:** Spore observations and removal of corrupted data for our analysis

The near-ground atmospheric spore measurements were made using a solar powered Burkard 7-day volumetric spore sampler (Burkard Scientific, Uxbridge, UK). The measurement periods coincided with the observed appearance of pathogen ascomata (fruit bodies) during late summer. Spore counts were then estimated using a light microscope later confirmed by real-time PCR (polymerase chain reaction; see Timmermann et al. (2011) and Hietala et al. (2013) for a full description of methods).

As indicated in Section 2 of the main text, one of the first steps of our analysis was to process the spore observations from Hietala et al. (2013) to remove days with missing or corrupted data within the peak diurnal emission period (see Table A1). Note that the proportion of daily spores emitted before and after the peak emission period is small (approximately 5%).

Days with an ill-defined emission peak (see Table A1) generally correspond to the start/end of the emission period, when total spore emissions are low. When considering *t_peak_*, these seven days were removed from the analysis.

| **Table A1:** Spore observation days and data processing | | | |
| --- | --- | --- | --- |
|  | Month | | |
| Year | July | August | September |
| 2009 | 29, 31 | 2, 5, 7, 9, 12, 17, 19, 21, 26 | 2, 9^#^, 11, 13^#^, 15, 16, 22^#^ |
| 2010 | [2–24], 26, 29, [31] | 2, 4, 7, 9, 12, 26*, 29* | 1*, 5*, 12*^#^, 19*^#^, 26*^#^ |
| 2011 | [4–25] |  |  |
| Notes: square brackets denote days and day ranges that have been excluded or partially excluded (for the case of day ranges) from the analysis. Days excluded from the day range in 2010 include the 9, 11, 12, 16 and 19 July. Days excluded from the day range in 2011 include the 18 and 19 July. Days were excluded due to missing or questionable data occurring close to the time of peak emission (close to sunrise). One common cause of data corruption was the germination of spores (i.e., the development of hyphae) on the adhesive spore tape used within the Burkard spore trap (see Hietala et al. 2013). The symbols # and * indicate days with ill-defined emission peaks and days with missing soil moisture data, respectively. The 10 July 2010 (within a day range) also had an ill-defined emission peak. | | | |

**APPENDIX B:** Diurnal change of hourly spore emissions and the averaging procedure to derive our simple Gaussian approximation of these observations


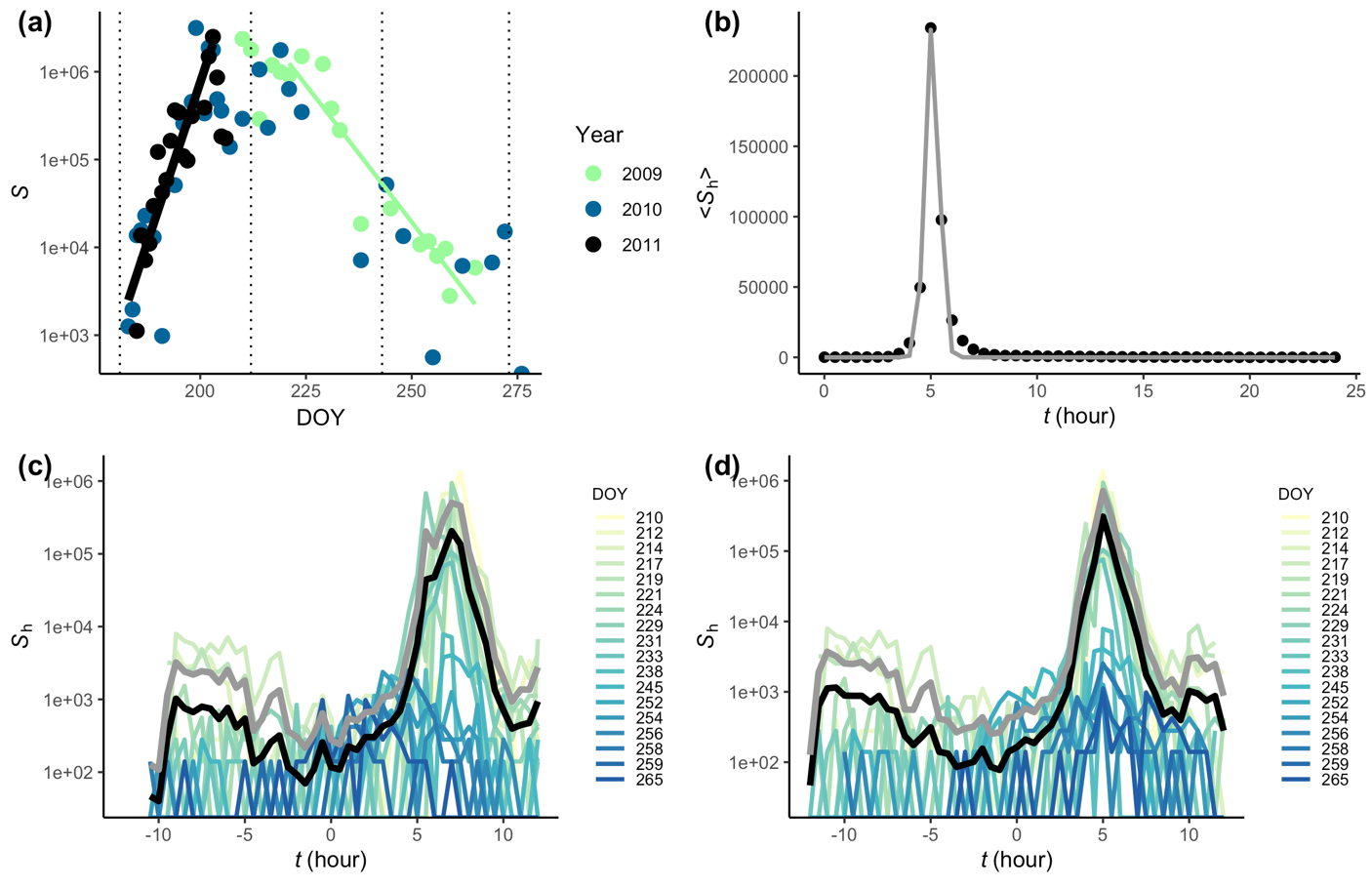


**Figure B1**: Plot **(c)** shows the diurnal variations of *H. fraxineus* near-ground atmospheric spore counts using data from Hietala et al. (2013) (number of spores h^−1^) for the 2009 dataset, denoted by *S*_h_, which reveals the natural variation in the spore emissions, and **(d)** shows the same data as **(c)** but after the data has been temporally shifted. Before averaging we temporally shifted each diurnal dataset so that each daily maximum emission coincided with the average maximum emission time. The black and grey curves in **(c)** and **(d)** show the mean and standard deviation of the data, respectively. The colour scale in **(c)** and **(d)** reveals the general reduction in emissions through late summer, as expected from Figure 1 **(a)** in the main text. Note that temporally shifting the data allowed the Gaussian to reveal the average shape of the emission peak. Otherwise the Gaussian is broadened by the variation in the maximum emission times.

**APPENDIX C:** A simple analytical model for the average diurnal variation of spore emissions which vary through the season

This appendix gives the details of Eq (1) in Section 2.1.

In order to derive a simple analytical expression for the average diurnal variation of spore emissions that vary through the season, we fit models by maximum likelihood and then consider only the deterministic part of those models. In other words all model residuals have been neglected.

The analytical seasonal model for the total daily spore counts is given by

$$S^{'}=\left\{ \begin{aligned} e^{L_{1}}, 183\leq d\leq203 , \\ L_{2} , 203 <d<221, \\ e^{L_{3}}, 221\leq d\leq265, \end{aligned} \right.$$

where *L_1_*, *L_2_* and *L_3_* are simple linear functions of the form *L = a0 + a1 d*, where *d* is the day of the year and *a0* and *a1* are constants. The linear models *L_1_* and *L_3_* were fitted to the log-transformed total daily spore counts for time periods 2 to 17 July, and 9 September to 22 October. The inequalities were chosen to capture the seasonal increase and decrease of spore emissions such that the models’ assumptions of normality and homogeneity of variance were met. The model parameters and summary statistics for these models are given in Table C1. The model *L_2_* links the other two models and has the form

$L_{2}={max(e}^{L_{1}})+\frac{{max(e}^{L_{3}})-{max(e}^{L_{1}})}{N}(d-d_{s})$,

where *N* is the number of days associated with *L_2_*, and *d_s_* is the last day of the year associated with *L_1_* (i.e. day of year 203). During the time period for *L_2_* the daily spore emissions were considered to be fairly constant and so a simple linear function was used rather than fitting a model to data. Since we are dropping the residuals anyway this seems reasonable.

The average diurnal variation of spore emissions ⟨*S_h0_*⟩, plotted in Figure B1(d), were fitted to a Gaussian model of the form

$$G(h)=Ce^{-\frac{{(h-\mu)}^{2}}{2\sigma^{2}}}\cong\left\langle S_{h0} \right\rangle,$$

where the model residuals have been neglected, *h* is the hour of the day, and *C* = max(⟨*S_h0_*⟩). The parameters (constants) *μ* and *σ* are the average time of the peak spore emission and the average standard variation of *μ*. Before fitting the Gaussian model we first shifted each diurnal dataset so that each day’s maximum emission coincided with the average maximum emission time. This allowed the model fit to reveal the average shape of the peak emission. If the data is not shifted in this way then the Gaussian is broader due to the natural variation of the maximum emission times. We then averaged the hourly emissions across all days and years. See Table C1 for the model parameters and summary statistics.

If we integrate the last equation across 24 hours then we have an average total daily emission. If we replace this average by *S’* then we find

$$C=\frac{S^{'}}{I}, I=\int_{0}^{24} e^{-\frac{\left( h-\mu\right)^{2}}{2\sigma^{2}}} dh=constant,$$

$${S^{'}}_{h}(d,h)=\frac{S'(d)}{I}e^{-\frac{\left( h-\mu\right)^{2}}{2\sigma^{2}}}$$

where we have assumed that *μ* and *σ* do not change through the season. Note that since *d* and *h* are both functions of *t*, the continuous time unit, we could find *S’_h_* (t). However, this would complicate the application of the model, for example, by using a more complex time unit such as Julian Days. In contrast it is straightforward to use day-of-year and hour of the day.

| **Table C1:** Seasonal and diurnal model parameters and summary statistics | |
| --- | --- |
| *S’*(d) | *G*(h) |
| Increasing spore emissions:  *a0* = −53.6, *a1* = 0.336,  *σ_a0_* = 5.793, *σ_a1_* = 0.030, *σ_m_* = 1.039,  χ^2^ = 33.46, *ν* = 31,  $\bar{r}^{2}$ = 0.80, F-statistic p-value: 2.0x10^-12^ | *C* = 2.383e+05 h^−1^, *μ* = 5.071 h,  *σ* = 0.327 h,  *σ_C_* = 4375 h^−1^,  *σ_μ_* = 8.379e-03 h, *σ_σ_* = 6.667e-03 h,  *σ_m_* = 4141,  χ^2^ = 1764, *ν* = 46,  $\bar{r}^{2}$ = 0.83, F-statistic p-value: 1.065e-13 |
| Decreasing spore emissions:  *a0* = 45.8, *a1* = −0.144,  *σ_a0_* = 3.704, *σ_a1_* = 0.015, *σ_m_* = 0.781,  χ^2^ = 6.72, *ν* = 11,  $\bar{r}^{2}$ = 0.88, F-statistic p-value: 1.3x10^-6^ |  |

**APPENDIX D:** Model fit and predictive ability across 1000 random data partitions


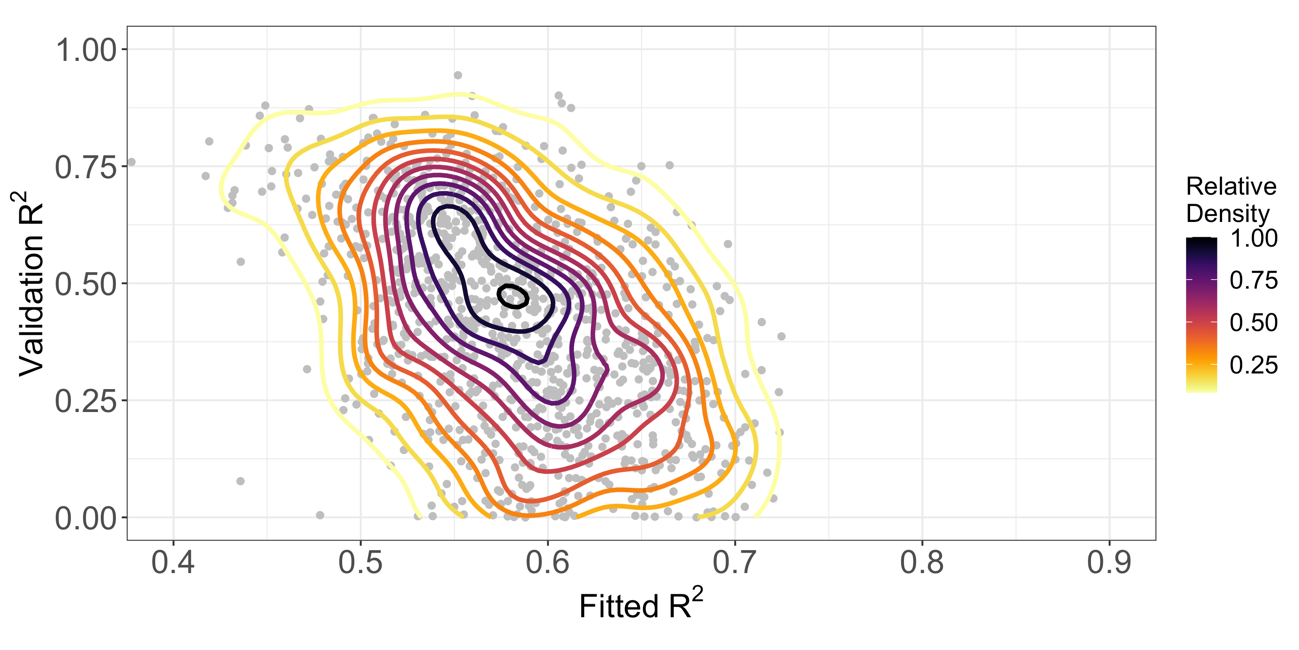


A


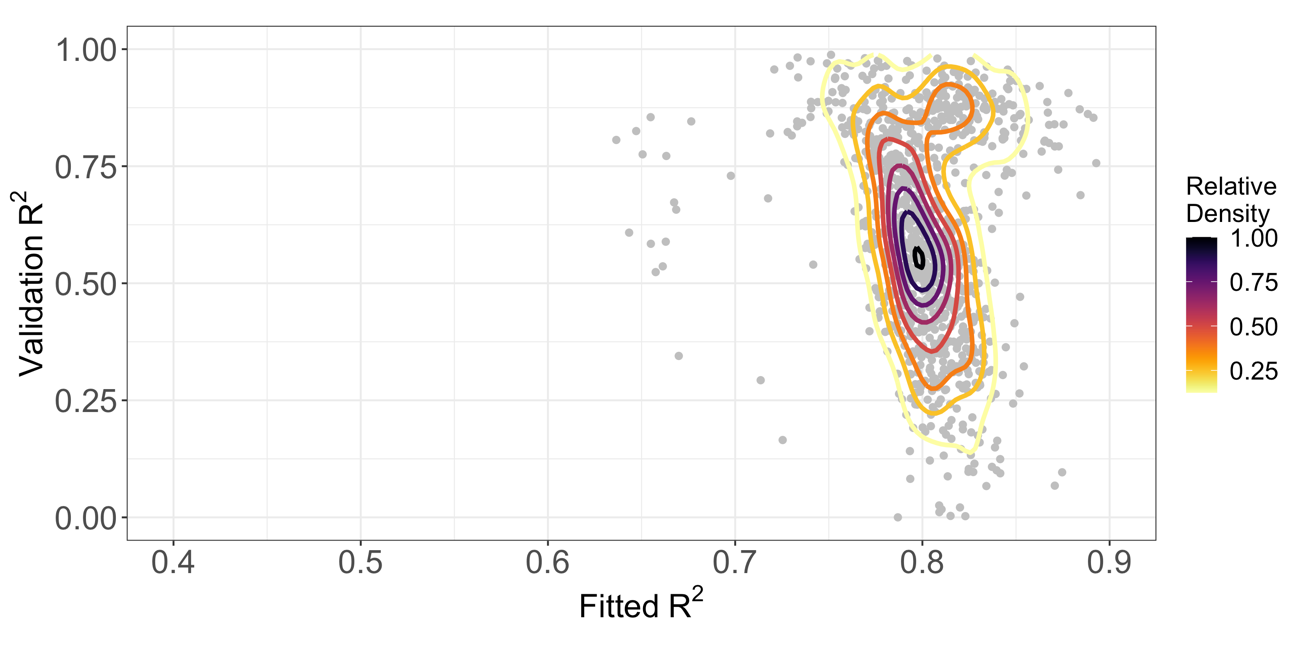


B

**Figure D1**: The R^2^ values (grey dots) across 1000 random data partitions for models of A) daily spore emissions, log_10_(*S*) and B) the time of peak spore emissions, *t*_peak_. The R^2^ values are for the data used to fit the model (x-axis) and to an independent validation dataset (y-axis). Contours show the relative density of the points (relative to the maximum estimated density of points).

**APPENDIX E:** Relative density of predicted marginal relationships


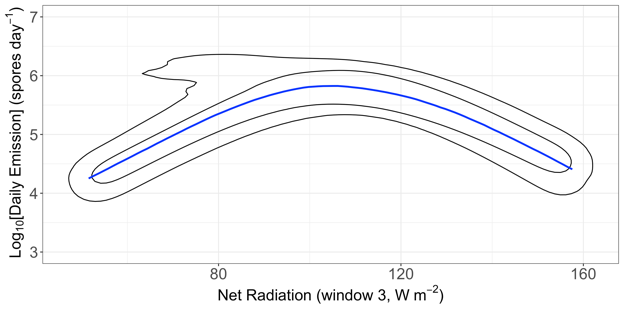

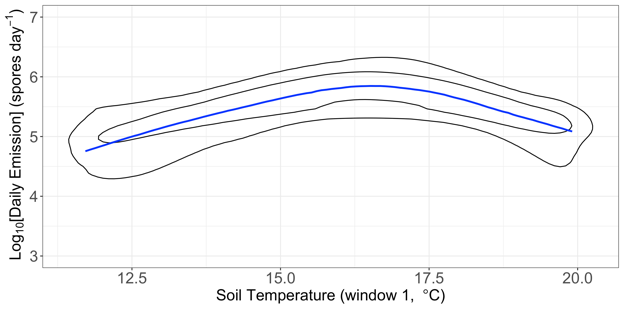


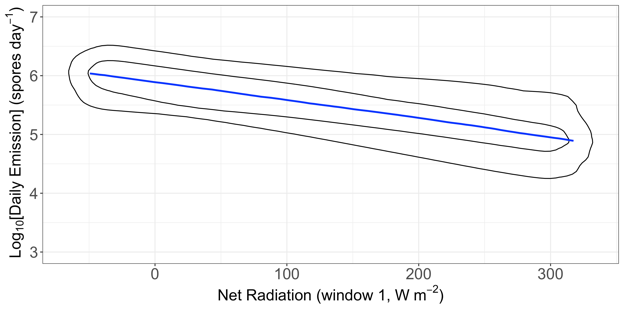

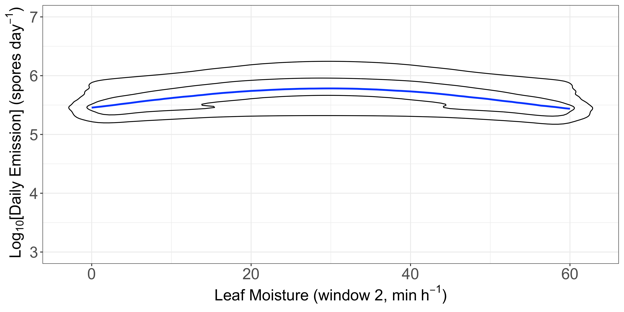


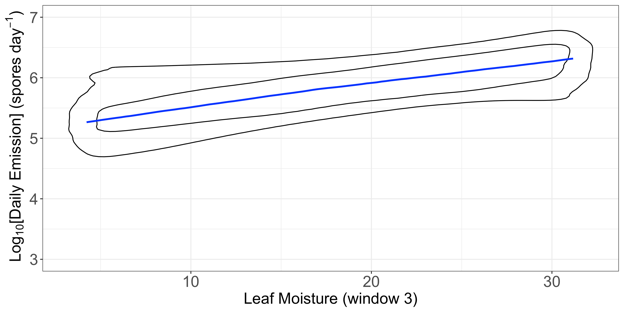

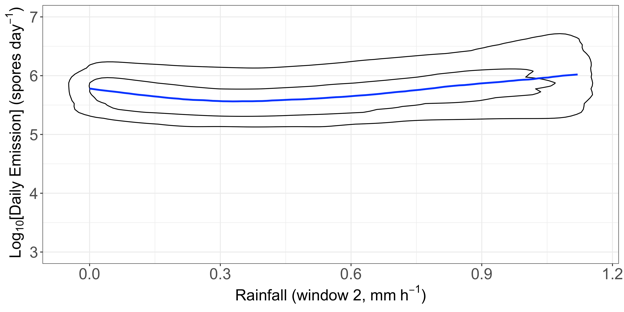


**Figure E1**: Relative density of predicted marginal relationship for daily total spore emission (similar to Figure 2 in main text) calculated across all 1000 models for the top six variables in Table 3. The inner and outer contours (black lines) are relative densities of 0.5 and 0.1 respectively (relative to the maximum density of predictions). The median prediction (across all 1000 models) is shown as a blue line.

**
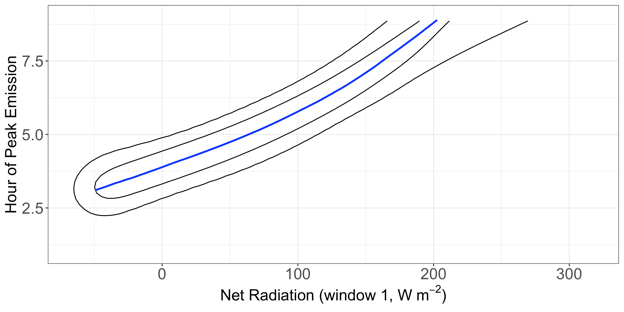

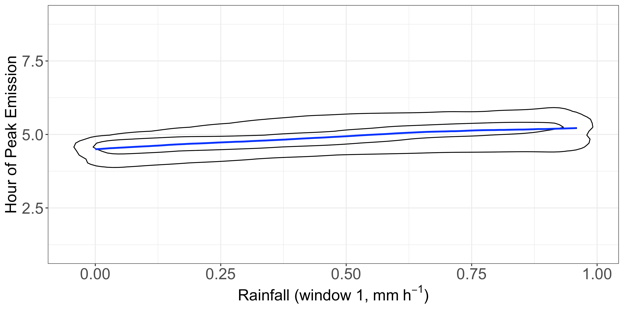
**

**
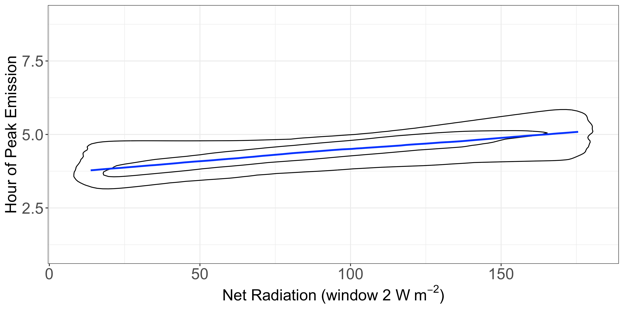

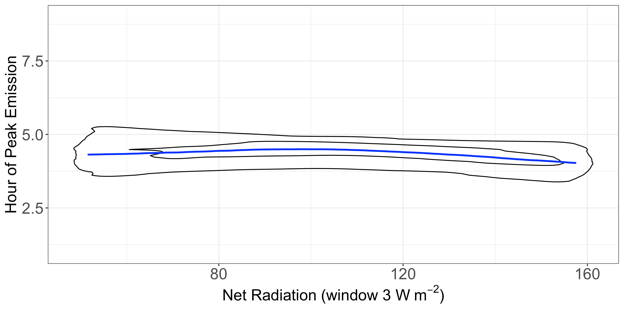
**

**Figure E2:** Relative density of predicted marginal relationship for timing of peak emission (similar to Figure 3 in main text) averaged across all 1000 models for the top four variables in Table 5. The inner and outer contours (black lines) are relative densities of 0.5 and 0.1 respectively (relative to the maximum density of predictions). The median prediction (across all 1000 models) is shown as a blue line.

**APPENDIX F:** Fitting models to 2009 and 2010 data including soil moisture as a covariate

We exclude soil moisture from the results in the main text in order to maximise the data available, because reliable soil moisture data was absent for 2011 and for part of 2010. However, soil moisture may be expected to have a role in spore emissions, so here we give the fitted models including soil moisture as a covariate. The models are fitted using the same methodology as in the main text using covariates shown in Table F1. The models are fitted to data from 2009 and 2010 (where there is soil moisture data).

| **Table F1**: The 10 meteorological variables, their three averaging windows and their use in the models for *t*_peak_ and log_10_(*S*). Squares marked - are variables that were excluded *a priori* based on the physics of the system*.* Variables used in the model for time of maximum spore emission are indicated with a *t*_peak_. Variables used in the model for total daily spore emissions are indicated with log_10_(*S*). Squares marked X indicate variables that were excluded due to colinearity. | | | | | |
| --- | --- | --- | --- | --- | --- |
|  |  |  | **Averaging window length** | | |
| **Variable name**  **& symbol** | **Units** | **Measurement details** | **window 1 (± 2 h)** | **window 2 (1 day)** | **window 3**  **(5 days)** |
| Rainfall, *R* | mm h^-1^ | RR, (1),  ±0.1 mm/h | *t*_peak_,  log_10_(*S*) | X | X |
| Soil moisture, $\theta$ | 1 | VAN1, (2), first 10 cm,  Volumetric, ±0.02 | *t*_peak_,  log_10_(*S*) | X | X |
| Leaf moisture, *M_s_* | min/h | BTff, (1), ∼2-m a.g.l., accuracy unknown. | *t*_peak_,  log_10_(*S*) | *t*_peak_  X | *t*_peak_,  log_10_(*S*) |
| Relative humidity, *U* | 1 | UMf, (1), hourly mean, ±2% (0 to 90%) | - | - | - |
| Soil temperature, *T_g_* | ^o^C | TJM1, (2), 1-cm deep, hourly mean, ±0.2 K | X | *t*_peak_,  log_10_(*S*) | X |
| Surface  temperature, *T_s_* | ^o^C | TS, (2), hourly mean,  ±0.2 K | - | - | - |
| Air temperature, *T_a_* | ^o^C | TMf, (1), 2-m a.g.l.,  hourly mean,  ±0.2 K | - | - | - |
| Net radiation, *R_n_* | W m^-2^ | RN, (2), surface flux, hourly mean, < 10% of each days integrated radiation. | *t*_peak_,  log_10_(*S*) | *t*_peak_,  log_10_(*S*) | X  log_10_(*S*) |
| Wind speed, *\|*u*\|* | m s^-1^ | FM2, (2), 2-m a.g.l.,  hourly mean,  horizontal speed,  1% ±0.1 m/s | *t*_peak_,  log_10_(*S*) | *t*_peak_,  log_10_(*S*) | - |
| Frost, *F* | presence/absence |  | - | *t*_peak_,  log_10_(*S*) | - |
| Notes: Acronyms within ‘Measurement details’ are the NIBIO variable names. Locations of observations are specified using the numbers in brackets, where 1 = Åsbakken and 2 = Ås, and equipment error estimates are provided. We derived an additional variable for the presence of frost when surface temperature is less than 0 ºC and leaf moisture is non-zero. Leaf moisture was observed using a leaf wetness sensor, which gives the time in minutes the leaf is wet within each hour (see Campbell Scientific, 2020 for more information). | | | | | |

| **Table F2**: The frequency (across 1000 fitted models to random data partitions) with which different model complexities (i.e. number of terms) were selected and their median R^2^ for the validation data. | | | | | | | | |
| --- | --- | --- | --- | --- | --- | --- | --- | --- |
| Number of smooth terms | 2 | 3 | 4 | 5 | 6 | 7 | 8 | 9 |
| **log_10_(S) Model** |  |  |  |  |  |  |  |  |
| Frequency | 2 | 97 | 349 | 340 | 158 | 45 | 7 | 2 |
| Median validation R^2^ | 0.20 | 0.55 | 0.64 | 0.58 | 0.54 | 0.51 | 0.32 | 0.28 |
|  |  |  |  |  |  |  |  |  |
| ***t_peak_* Model** |  |  |  |  |  |  |  |  |
| Frequency | 1 | 23 | 156 | 323 | 322 | 150 | 23 | 2 |
| Median validation R^2^ | 0.72 | 0.46 | 0.52 | 0.57 | 0.55 | 0.55 | 0.44 | 0.38 |

| **Table F3**: The variables and time-scale for the 11 smooth terms in the GAM model for log_10_(*S*) (Table F1) and the proportion of the 1000 randomisations for which each term was selected in the final model. Terms selected in more than 70% of models are highlighted in bold. | | |
| --- | --- | --- |
| **Variable** | **Time-scale** | **Selection**  **Proportion** |
| **Net radiation** | **Window 3** | **1.00** |
| **Leaf moisture** | **Window 3** | **0.85** |
| **Rainfall** | **Window 2** | **0.83** |
| **Net radiation** | **Window 2** | **0.78** |
| Leaf moisture | Window 1 | 0.36 |
| Rainfall | Window 1 | 0.28 |
| Wind speed | Window 2 | 0.25 |
| Wind speed | Window 1 | 0.13 |
| Soil temperature | Window 2 | 0.12 |
| Soil moisture | Window 1 | 0.11 |
| Net radiation | Window 1 | 0.03 |

| **Table F4:** The variables and time-scale for the 11 smooth terms in the GAM model for t_peak_ (Table F1) and the proportion of the 1000 randomisations for which each term was selected in the final model. Terms selected in more than 70% of models are highlighted in bold. | | |
| --- | --- | --- |
| **Variable** | **Time-scale** | **Selection**  **Proportion** |
| **Soil moisture** | **Window 1** | **1.00** |
| **Net radiation** | **Window 1** | **1.00** |
| Rainfall | Window 1 | 0.69 |
| Leaf moisture | Window 2 | 0.67 |
| Leaf moisture | Window 1 | 0.61 |
| Net radiation | Window 2 | 0.58 |
| Net radiation | Window 3 | 0.46 |
| Wind speed | Window 1 | 0.30 |
| Leaf moisture | Window 3 | 0.11 |
| Wind speed | Window 2 | 0.11 |
| Soil temperature | Window 2 | 0.09 |


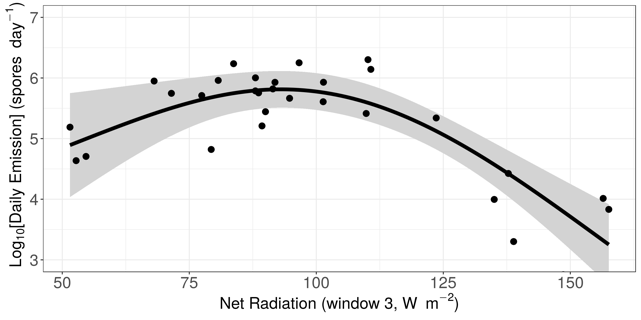

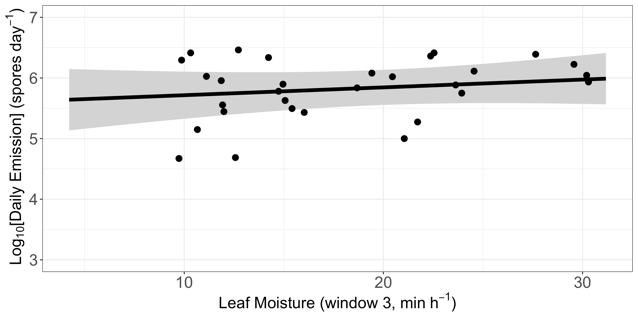


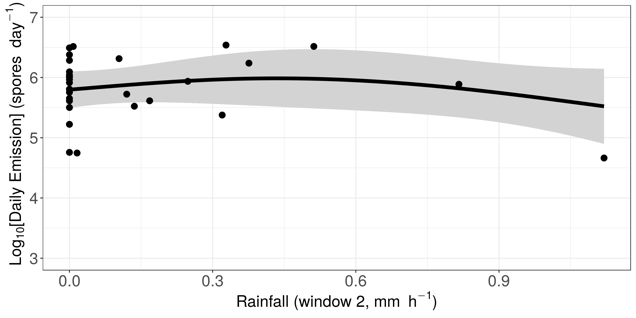

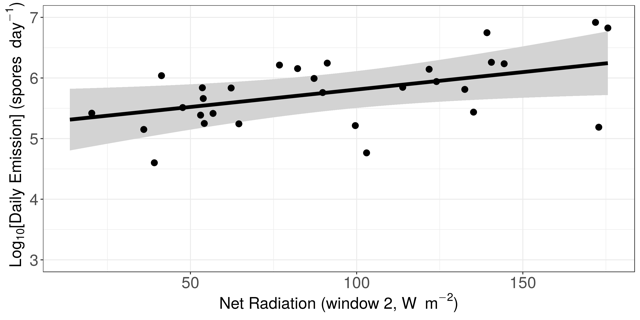


**Figure F1**: The marginal predictions (black line) and 95% confidence intervals (grey region) from the most common model for total daily spore emissions (four smoothed terms). Residuals are shown by solid circles. Predictions are calculated at the median values for the remaining three variables.


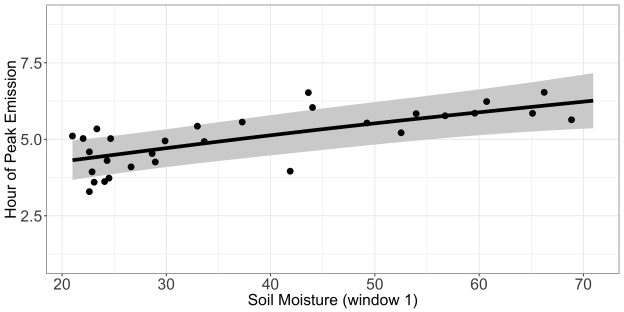

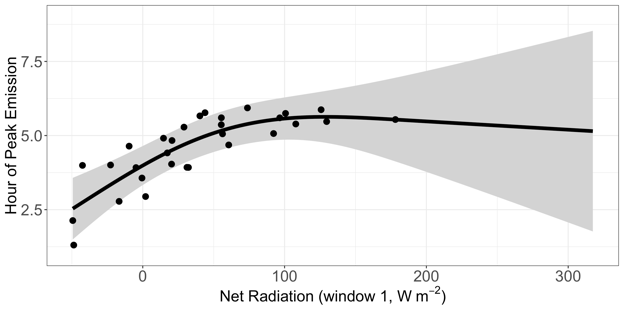


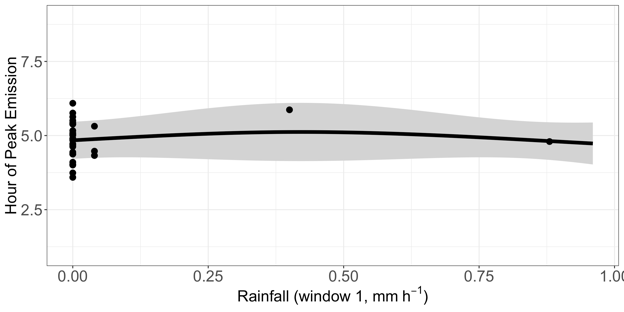

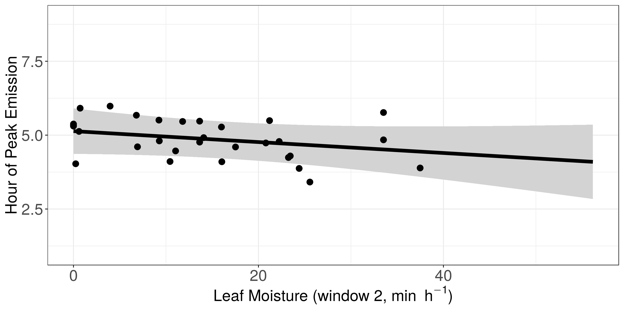


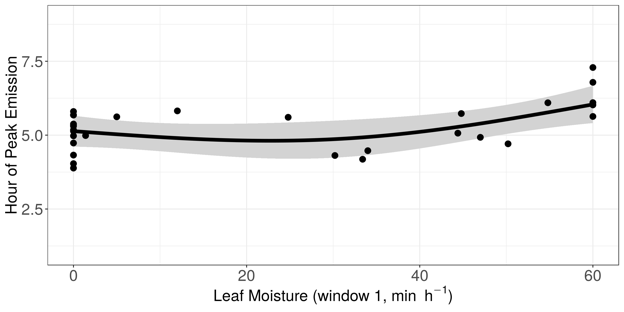

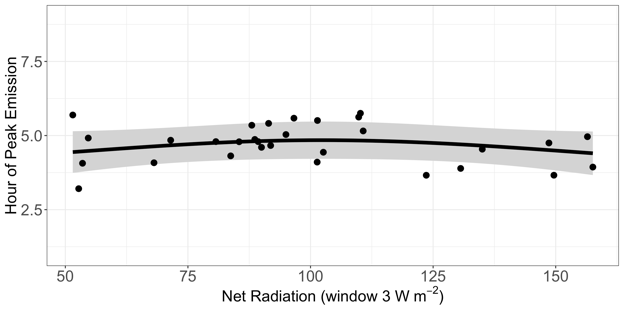


**Figure F2:** The marginal predictions (black line) and 95% confidence intervals (grey region) from the most common model of timing of peak spore emissions (six smoothed terms). Residuals are shown by solid circles. Predictions are calculated at the median values for the remaining five variables.

**APPENDIX G:** Random Forest Analysis of Spore Emission Data

**Methods**

Two random forests, each containing 1000 regression trees, were built to predict log_10_(*S*) and the *t*_peak_. The random forests were trained using data from 2010 and 2011. Data from 2009 was used as an independent test data set. Eleven meteorological variables were used as independent variables (Table 1 of main manuscript). Each random forest was built using 37 observations (approximately 80% of the data from 2010 and 2011), randomly sampled without replacement. The remaining nine observation were used as an out-of-bag sample for error estimation. This out-of-bag error estimate is almost identical to N-fold cross validation (Hastie et al 2009). Each tree was built using three randomly selected variables to build each node and a minimum of three training data points in any terminal node of a tree.

The performance of each random forest was quantified using both the out-of-bag sample and the independent test data. Performance was quantified using the mean squared error and the percentage variance explained. A variable’s importance was quantified using the out-of-bag sample as the average (across all 1000 trees) percentage increase in the mean squared error when the variable in the out-of-bag sample was permuted.

The models were fitted using the randomForest package (Liaw and Weiner, 2002) in R version 4.1.1 (R Core Team, 2021)

**Results**

The random forest for spore concentrations, log_10_(S), had an out-of-bag mean squared error of 0.56 and explained 29% of the variance in log_10_(S). The testing data from 2009 produced a mean squared error of 0.74 with 22% of the variance in log_10_(S) being explained (Figure G1). The predictive performance of the random forest was less than half of the approach using GAMs (Table G1). Variable importance gave five variables as the most important at predicting daily spore counts (Figure G2). These variables agreed well with the original GAM modelling approach, where four of the top five important variables were identified by both approaches (Table G2).

The random forest for the timing of peak daily spore counts, *t*_peak_, had an out-of-bag mean squared error of 3.1 h and explained 40% of the variance in *t*_peak._ The testing data from 2009 produced a mean squared error of 3.1 h with 2% of the variance in *t*_peak_ being explained (Figure G3). The predictive performance of the random forest was an order of magnitude less than the approach using GAMs (Table G1). Variable importance gave one variable as the most important at predicting *t*_peak_ (Figure G4). This variable was also identified as the most important by the original GAM modelling approach (Table G3).

**Conclusions**

The random forest approach has many similarities with the GAM approach. Both approaches use an ensemble of models (here 1000 models), both provide measures of predictive performance and variable importance, both provide predictions on independent test data. The variables identified as the most important explanatory variables are broadly consistent.

The difference between the two approaches is in their ability to model the shape of the relationship between the response variable and the meteorological explanatory variables. The random forest has less predictive ability (in the case of t_peak_ this is an order of magnitude less). This could be due to the small data set, since random forests are usually (but not always) applied in situations with large sample sizes.

Based upon these results we decide to use the GAM modelling approach as the main approach, and present the random forest in supplementary material.

| **Table G1:** The percentage of variance in the response explained by random forest and GAM modelling approaches using independent data from 2009. | | |
| --- | --- | --- |
| **Response** | **Random Forest** | **GAM** |
| log_10_(S) | 22% | 58% |
| *t*_peak_ | 2% | 37% |

| **Table G2:** The ranks of the variable importance for models of daily spore counts, log_10_(S), from random forest and GAM modelling approaches. The frost variable is excluded from the table because it was present *a priori* in all GAM models. | | |
| --- | --- | --- |
|  | **Rank of Variable Importance** | |
| **Variable** | **Random Forest** | **GAM** |
| Leaf moisture (5 day) | 1 | 5 |
| Net Radiation (5 day) | 2 | 1 |
| Net Radiation (1 day) | 3 | 7 |
| Soil Temp (1 day) | 4 | 2 |
| Net radiation (2 h) | 5 | 3 |
| Rainfall (1 day) | 6 | 6 |
| Leaf moisture (2 h) | 7 | 4 |
| Rainfall (2 h) | 8 | 9 |
| Wind (2 h) | 9 | 8 |
| Wind (1 day) | 10 | 10 |

| **Table G3:** The ranks of the variable importance for models of time of daily peak spore count, *t*_peak_, from random forest and GAM modelling approaches. The frost variable is excluded from the table because it was present *a priori* in all GAM models. | | |
| --- | --- | --- |
|  | **Rank of Variable Importance** | |
| **Variable** | **Random Forest** | **GAM** |
| Net radiation (2h) | 1 | 1 |
| Wind (1 day) | 2 | 8 |
| Soil temp (1 day) | 3 | 6 |
| Leaf moisture (1 day) | 4 | 7 |
| Net radiation (1 day) | 5 | 3 |
| Leaf moisture (2 h) | 6 | 5 |
| Rainfall (2 h) | 7 | 2 |
| Wind (2 h) | 8 | 10 |
| Net radiation (5 day) | 9 | 4 |
| Rainfall (5 day) | 10 | 9 |


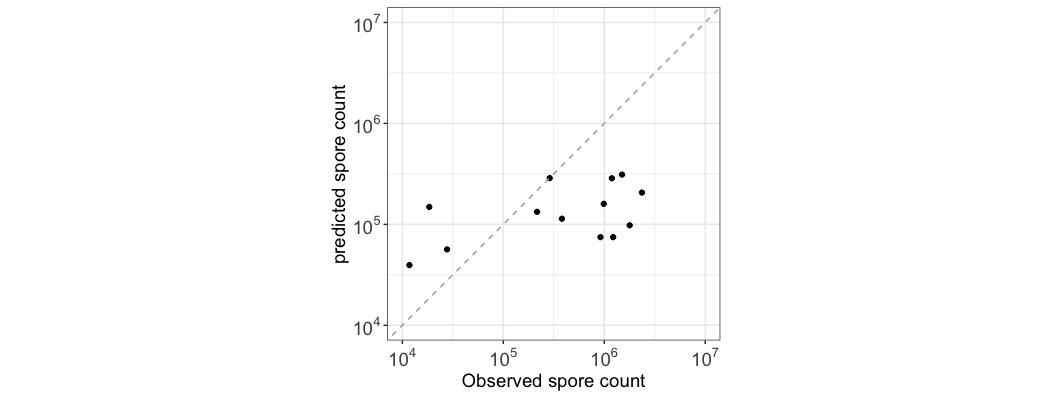


**Figure G1:** Observed daily spore counts for 2009 versus predictions from the random forest model (R^2^=22%). Dashed diagonal line represents observed equal to predicted.


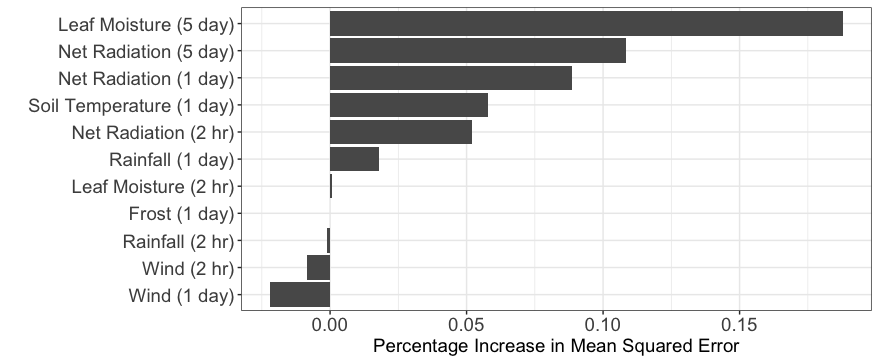


**Figure G2:** The importance of the 11 meteorological variables for the random forest model of daily spore counts, log_10_(S). A variable’s importance is measured by the percentage increase in the mean squared error


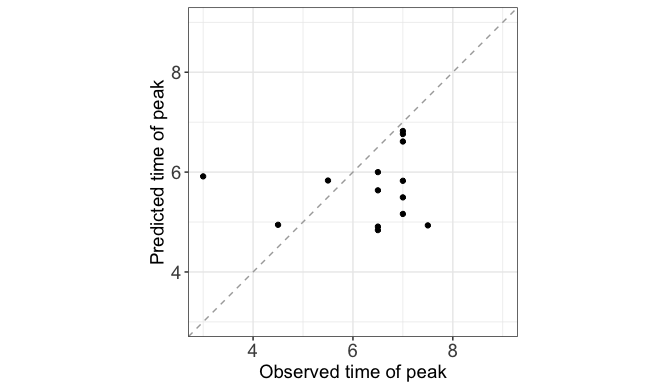


**Figure G3:** Observed timing of the daily peak in spore counts (hours since midnight) for data from 2009 versus predictions from the random forest model (R^2^=2%). Dashed diagonal line represents observed equal to predicted


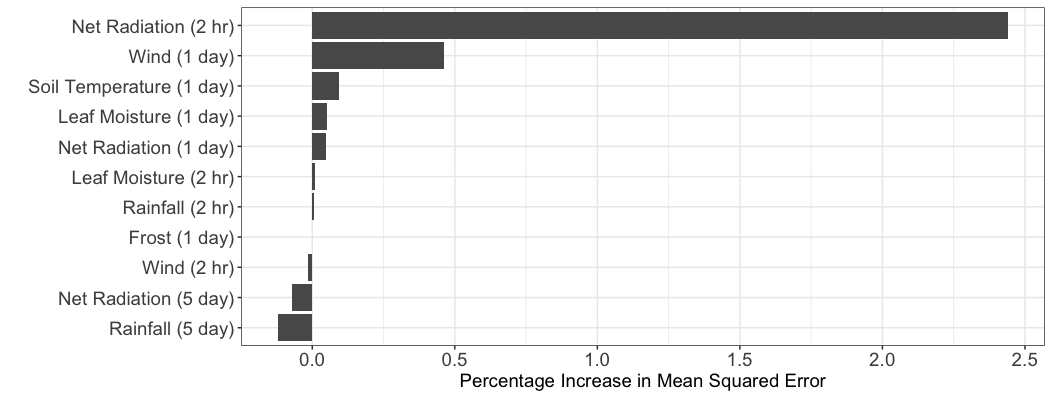


**Figure G4:** The importance of the 11 meteorological variables for the random forest model of timing of peak spore count, *t*_peak_. A variable’s importance is measured by the percentage increase in the mean squared error

**References**

Hastie, T., Tibshirani, R., Friedman, J., 2009. The Elements of Statistical Learning (2^nd^ Edition), Springer (New York).

Liaw, A., Wiener, M., 2002. Classification and Regression by randomForest. R News 2(3), 18--22.

R Core Team, 2021. R: A language and environment for statistical computing. R Foundation for Statistical Computing, Vienna, Austria. URL https://www.R-project.org/.
